# Decoding the genetic relationship between Alzheimer’s disease and type 2 diabetes: potential risk variants and future direction for North Africa

**DOI:** 10.3389/fnagi.2023.1114810

**Published:** 2023-06-05

**Authors:** Wided Boukhalfa, Haifa Jmel, Nadia Kheriji, Ismail Gouiza, Hamza Dallali, Mariem Hechmi, Rym Kefi

**Affiliations:** ^1^Laboratory of Biomedical Genomics and Oncogenetics, Institut Pasteur de Tunis, Tunis, Tunisia; ^2^Tunis El Manar University, Tunis, Tunisia; ^3^Faculty of Medicine of Tunis, Tunis, Tunisia; ^4^University of Angers, MitoLab Team, Unité MitoVasc, UMR CNRS 6015, INSERM U1083, SFR ICAT, Angers, France

**Keywords:** pathogenic variants, pathways, ethnicity, PRISMA, admixure, multidimensional scaling plot, miRNA

## Abstract

**Introduction:**

Alzheimer’s disease (AD) and Type 2 diabetes (T2D) are both age-associated diseases. Identification of shared genes could help develop early diagnosis and preventive strategies. Although genetic background plays a crucial role in these diseases, we noticed an underrepresentation tendency of North African populations in omics studies.

**Materials and methods:**

First, we conducted a comprehensive review of genes and pathways shared between T2D and AD through PubMed. Then, the function of the identified genes and variants was investigated using annotation tools including PolyPhen2, RegulomeDB, and miRdSNP. Pathways enrichment analyses were performed with g:Profiler and EnrichmentMap. Next, we analyzed variant distributions in 16 worldwide populations using PLINK2, R, and STRUCTURE software. Finally, we performed an inter-ethnic comparison based on the minor allele frequency of T2D-AD common variants.

**Results:**

A total of 59 eligible papers were included in our study. We found 231 variants and 363 genes shared between T2D and AD. Variant annotation revealed six single nucleotide polymorphisms (SNP) with a high pathogenic score, three SNPs with regulatory effects on the brain, and six SNPs with potential effects on miRNA-binding sites. The miRNAs affected were implicated in T2D, insulin signaling pathways, and AD. Moreover, replicated genes were significantly enriched in pathways related to plasma protein binding, positive regulation of amyloid fibril deposition, microglia activation, and cholesterol metabolism. Multidimensional screening performed based on the 363 shared genes showed that main North African populations are clustered together and are divergent from other worldwide populations. Interestingly, our results showed that 49 SNP associated with T2D and AD were present in North African populations. Among them, 11 variants located in *DNM3*, *CFH*, *PPARG*, *ROHA*, *AGER*, *CLU*, *BDNF1*, *CST9*, and *PLCG1* genes display significant differences in risk allele frequencies between North African and other populations.

**Conclusion:**

Our study highlighted the complexity and the unique molecular architecture of North African populations regarding T2D-AD shared genes. In conclusion, we emphasize the importance of T2D-AD shared genes and ethnicity-specific investigation studies for a better understanding of the link behind these diseases and to develop accurate diagnoses using personalized genetic biomarkers.

## Introduction

The world is experiencing the oldest living population (United Nations Department of Economic and Social Affairs, Population Division, 2020). The increase in lifespan and unhealthy habits coincides with an increase in age-related diseases, such as dementia and type 2 diabetes (T2D) (Hayden, 2019). Globally, more than 57.4 million adults live with dementia, and this number is estimated to increase to 152.8 million by 2050 (Nichols et al., 2022). Similarly, 537 million T2D patients, and this figure is expected to increase to 780 million (Magliano et al., 2021).

Alzheimer’s disease (AD) is the most common form of dementia worldwide, accounting for more than 70% of all cases (2022 Alzheimer’s Disease Facts and Figures, n.d.). Currently, more than 6.2 million adults above the age of 65 years live with AD in the United States alone (2022 Alzheimer’s Disease Facts and Figures, n.d.). The increasing prevalence of AD imposes a heavy socioeconomic burden on families and societies (Shu et al., 2022). AD is a complex disease, and the absence of modifying treatments adds another constraint. Therefore, a shift from a curative to a preventive approach is essential (Silva-Spínola et al., 2022). One approach is to work on the causes and risk factors of disease. For example, genetic investigation of AD risk factors could help shape our understanding of the disease and provide a promising tool for identifying presymptomatic AD (de Rojas et al., 2021). Furthermore, T2D is a major risk factor for AD development (Thomassen et al., 2020), and compelling evidence supports the interaction between these diseases (Barbagallo and Dominguez, 2014). T2D and AD share several molecular mechanisms including insulin resistance, oxidative stress (De Sousa et al., 2020), inflammation, and mitochondrial dysfunction (Silzer and Phillips, 2018). Thus, well-established genetic variants and pathways that are common between T2D and AD are of great significance for AD prevention and early diagnosis.

Advances in omics technologies, such as Genomics and Transcriptomics, have greatly enhanced our knowledge of the pathophysiology of T2D and AD at a detailed molecular level (Karczewski and Snyder, 2018). Several omics results have paved the way for new findings regarding the interactions between these diseases.

Although omics technologies represent great promise for science revolution and precision medicine implementation, a vast number of omics research cohorts are of European ancestry (Popejoy and Fullerton, 2016). This could lead to a serious research gap since European ancestry findings do not necessarily replicate across other populations (Martin et al., 2019). Genetic background is an important element when studying common diseases, such as AD and T2D (Huang et al., 2017). Consequently, there is an urgent need to integrate more underrepresented populations to maximize the potential of discovering genes and pathways that are common between T2D and AD (Popejoy and Fullerton, 2016) and to fulfill the promise of precision medicine.

North African populations have highly diverse and complex genetic structures. It is characterized by a rich genetic background due to the admixture between Berber (early settlers in North Africa) and Eurasiatic and Sub-Saharan components (Kefi et al., 2015). Like a mosaic, the North African genetic background represents a valuable and unique source for genetic investigations (Ben Halima et al., 2017; Jmel et al., 2018; Arauna et al., 2019; Romdhane et al., 2021; Dallali et al., 2022) and the implementation of precision medicine.

In this study, we aimed to identify the most common variants and pathways shared between T2D and AD and to explore their genetic variability in North African populations compared to other populations worldwide.

## Materials and methods

**Figure 1:**
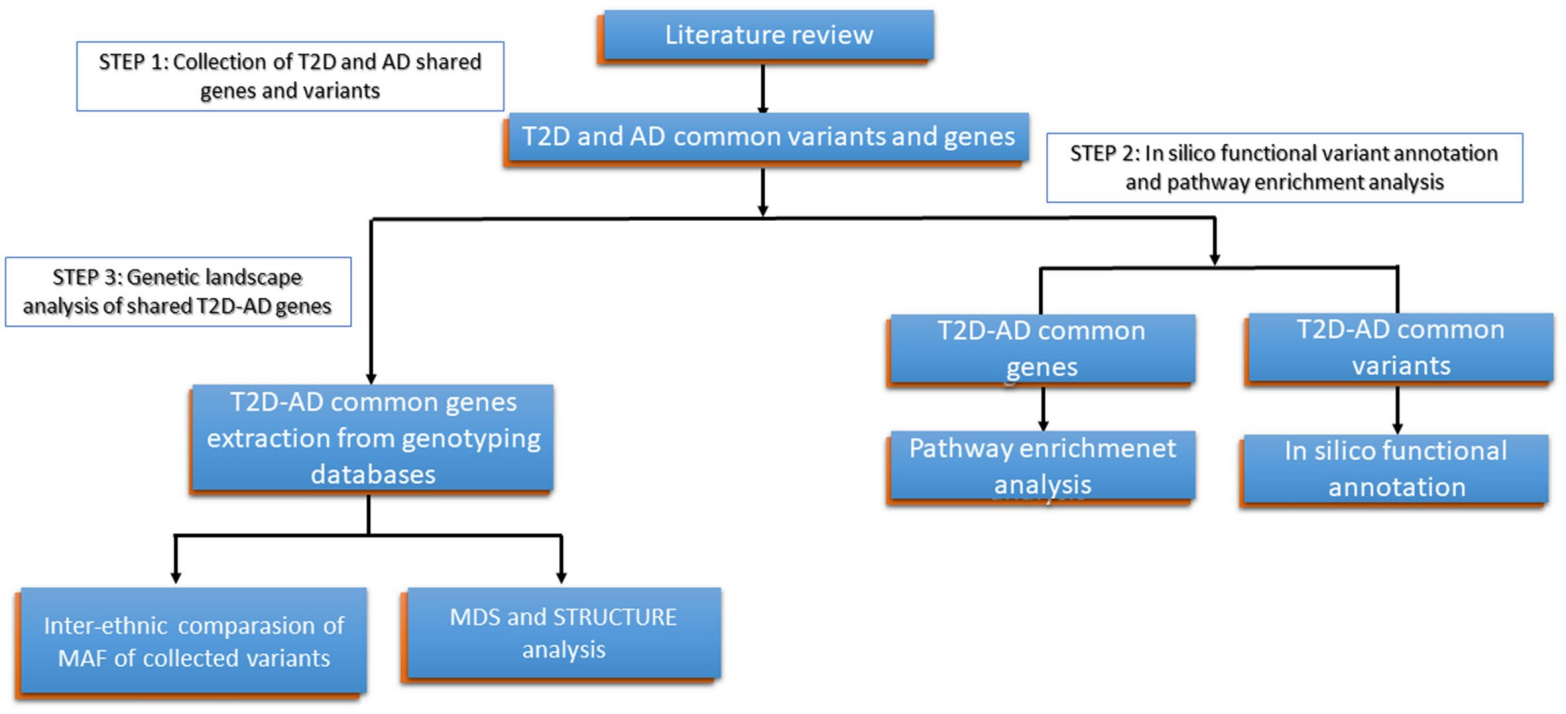
Detailed research work flow. Type 2 Diabetes (T2D); Alzheimer’s disease (AD), Minor allele frequency (MAF).

To attend our objectives, (1) we developed the present workflow (Figure 1), in which we conducted in the first step a general review of the literature to collect genes and variants previously identified in common between T2D and AD, (2) Then, in the second step, we performed *in silico* functional analysis and pathway enrichment analysis of the collected variants and genes shared betweenT2D and AD, and (3) In the third step, we conducted a multidimensional scaling plot (MDS) and Structure analysis of these variants on genotyping data available publicly in order to explore the genetic landscape of T2D-AD shared genes in North African populations and in comparison, to other populations.

### Step 1: T2D-AD shared genes and variants collection

To collect T2D-AD shared genes and variants from the literature, we developed a study protocol using the PRISMA statement (Page et al., 2021). The public database PubMed^1^ was searched from August 2001 to the 4th of September 2022. The search terms were limited to “Type 2 diabetes” AND “Alzheimer’s disease” AND “gene” OR “biomarker” OR “Proteomic” OR “Methylation.” Initially, we established a systematic screening for all articles published during that period according to their title and abstract relevance. Articles on animal models, *in vivo* studies, and mitochondrial DNA were excluded. The final selection criteria were as follows: (1) relevant articles, (2) available in English, (3) studies conducted on human samples, and (4) genetic, transcriptomic, proteomic, and methylation studies.

### Step 2: *In silico* functional variant annotation and pathway enrichment analysis

#### Variant annotation and functional effect prediction

The collected single nucleotide polymorphism (SNPs) from the selected studies were annotated using the VEP (Variant Effect Predictor) tool from Ensembl (McLaren et al., 2016) and the SNPnexus web server (Oscanoa et al., 2020). Next, we used two databases to annotate the functionalities of the variants, depending on their locations. Variants in the coding region have been functionally annotated using Polyphen-2 (Adzhubei et al., 2010). Polyphen-2 is a web-based software that can predict the possible impact of amino acid substitutions on the structure and function of human proteins using physical and evolutionary comparative considerations (Adzhubei et al., 2010). The PolyPhen2 scores range between 0 and 1, with 1 being the most likely deleterious variant. Similarly, RegulomeDB^2^ was used to prioritize non-coding and modifier variants. RegulomeDB is an open-access database that annotates variants in the intergenic region based on ENCODE releases, Gene Ontology, Chromatin States from the Roadmap Epigenome Consortium, and updates to DNase footprinting. The RegulomeDB probability score is ranging from 0 to 1, with 1 being the most likely to be a regulatory variant (Boyle et al., 2012). Top-ranked SNPs (RegulomeDB score = 1) were further investigated using the GTEx portal (Stanfill and Cao, 2021) and FeatSNP to assess their association with epigenetic effects in the human brain (Ma et al., 2019). Additionally, SNPinfo, a web-based server, was used to detect SNPs with potential miRNA-binding sites (Xu and Taylor, 2009). Variants with predicted effects on miRNA-binding sites were explored using miRdSNP (Bruno et al., 2012). The list of miRNAs was then used to generate a heat map of pathways affected by this miRNA using miRPathDB 2.0 (Kehl et al., 2020).

#### Pathway enrichment analysis and visualization

Pathway enrichment analysis is an efficient method for gaining mechanistic insight into a specific gene list by identifying biological pathways enriched in that gene set (Reimand et al., 2019). We performed pathway enrichment analysis for the replicated genes among studies using the g:Profiler tool (Raudvere et al., 2019). It searches a collection of gene sets representing Gene Ontology (GO) terms and pathways (KEGG pathway, Reactome, and WikiPathway). The Bonferroni correction was applied as the significance threshold for all enrichment analyses. The user threshold was set to 0.05. However, pathway enrichment analysis often highlights several versions of the same pathway (Reimand et al., 2019). Visualization tools can help facilitate the interpretation of analysis results. Hence, we used EnrichmentMap (Merico et al., 2010) to visualize the non-redundant pathways.

### Step 3: Genetic landscape analysis of T2D-AD shared genes

#### Genotyping data and quality control analysis

Genotyping data of 829 individuals from 16 populations were downloaded from the International 1,000 Genome Project phase III (1000 Genome, n.d)^3^ and published data (Li et al., 2008; Henn et al., 2012). The studied populations included those of American: African ancestry in the South Western USA (ASW) and people of Mexican ancestry living in Los Angeles, California, USA (MEX); European ancestry: Northwestern and Western European ancestry populations of Utah from the CEPH collection (CEU), Toscani people of Italy (TSI), South Spain (Spain_S), North Spain (Spain_N), North West of Spain (Spain_NW) and Spain Basic populations (Spain_BASC); individuals from East Asian ancestry: Han Chinese in Beijing, China (CHB), the Chinese population of metropolitan Denver, Colorado, USA (CHD) and Japanese in Tokyo, Japan; (JPT), Individuals from North Africa: Algeria (Algeria), Egypt (Egypt), Libya (Libya), Tunisia Douiret (TN_Ber), South Morocco (Morocco_S), North Morocco (Morocco_N).

We used the PLINK v2 software (Chang et al., 2015) to extract variants of the selected common genes between T2D and AD, from the genotyping data.

First, to study the genetic landscape of all variants (common and rare), we excluded variants deviating from the Hardy–Weinberg equilibrium (HWE) (*p*-value < 10^−4^) and those with a genotyping rate ≤ 95% for each of the studied populations. Second, we retrained variants with minor allele frequency (MAF) >10^−2^ to explore the genetic landscape of common variants given their importance in the development of complex diseases (de Rojas et al., 2021; Shoily et al., 2021).

### Statistical analysis

Merged data were pruned based on the physical distances between adjacent markers and linkage disequilibrium (LD). High-density markers that did not provide additional information were excluded. Next, pruning data were used to create a multidimensional scaling plot (MDS) to study the landscape of the selected common T2D-AD gene regions. To this end, a symmetric matrix of identity-by-state (IBS) distances for all pairs of individuals was based on the proportion of shared common alleles. This analysis was performed using the Plink and R software (R: The R Project for Statistical Computing, n.d).^4^

After calculating the allele frequencies of the T2D-AD shared variants, the populations were clustered according to their geographic origins. Four groups were generated: North African (NAF), East Asian (EAS), American (AMR), and European (EUR). The Chi-square test was used to compare the risk allele frequencies of candidate variants between NAF populations and other populations. Bonferroni’s adjustment was applied to the level of significance set at a value of p threshold of 5% divided by the number of studied variants. All analyses were conducted using the R software.

### Analyses of population genetic structures

We used a Bayesian clustering algorithm, STRUCTURE Ver. 2.3.4 software (Pritchard et al., 2000; FALUSH et al., 2007) to explore the variability of the common T2D and AD variants in terms of population structure. The algorithm assigns samples within a hypothetical K number of ancestries. We set a range of possible numbers of clusters ranging from *K* = 2 to *K* = 10, and four trials were run for each K. The Markov Chain Monte Carlo iteration for each structure analysis was run for 10,000 after an initial burn-in period of 10,000 steps. To assess the most likely number of clusters, we calculated Delta K, as proposed by Evanno et al. (2005). The similarity of the runs at each K level was evaluated using CLUMPP software as implemented online (Jakobsson and Rosenberg, 2007). Distruct software was used to visualize the best alignment of subpopulations, inferring population substructure and individual assignment across the best runs at each *K* level.

## Results

### Step 1: T2D-AD shared genes and variants collection

**Figure 2:**
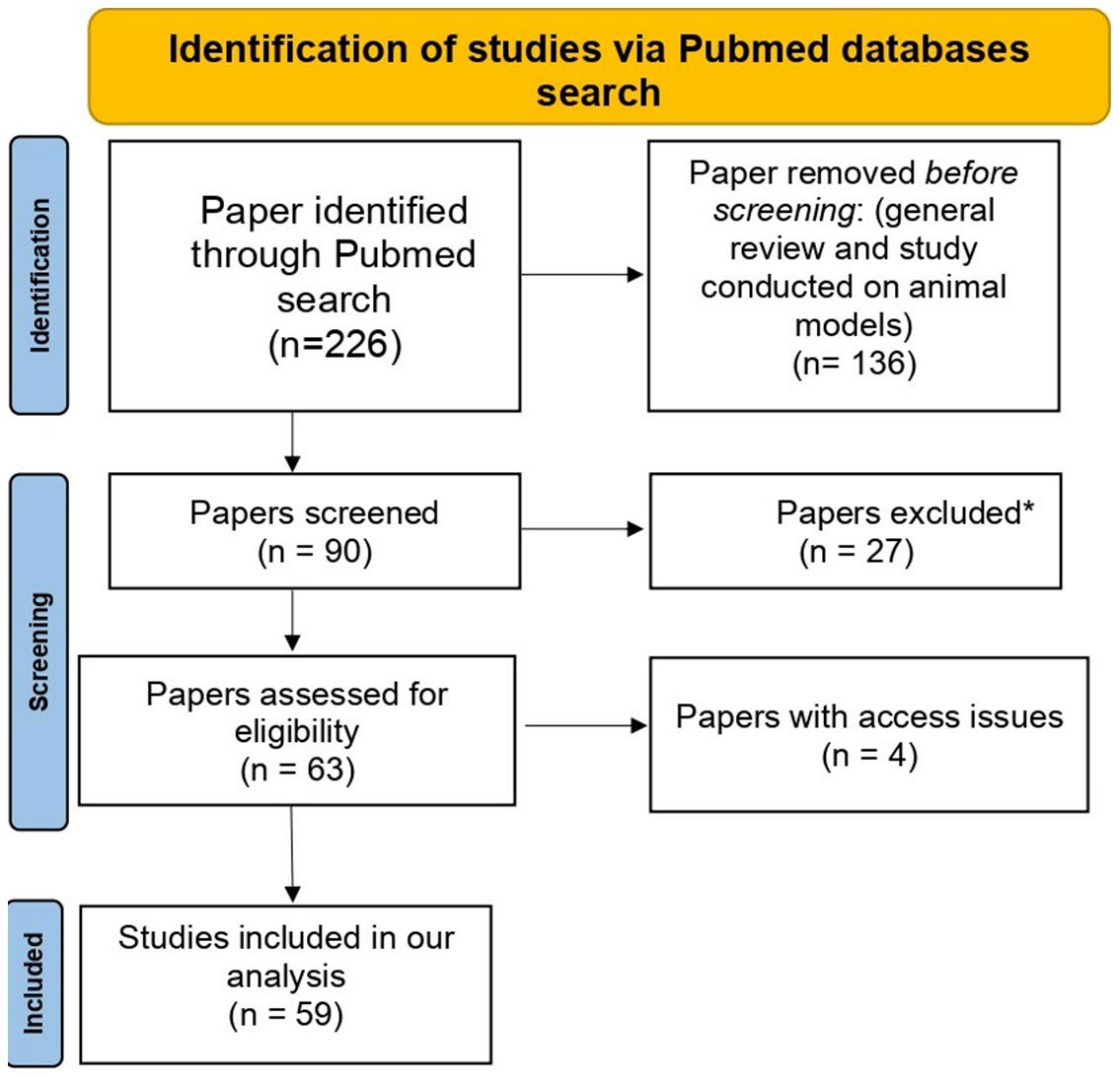
PRISMA flow diagram of selected studies. *Excluded papers are those with irrelevant results.

The PubMed search based on our defined search terms yielded 226 results. Studies with irrelevant results and those conducted using animal models (*n* = 163) were excluded. Additionally, four relevant studies were excluded due to access issues. The PRISMA flow diagram for the selected studies is represented in Figure 2. Finally, 59 publications were included in the present study (Supplementary file 1; Table 1).

The majority of the studies used data from American (Native American, Latino American, African American, and Mexican), Asian (Han Chinese, Japanese, and Korean), and European ancestry populations.

The literature search revealed 231 variants and 363 genes shared between T2D and AD identified by these studies. The 363 genes include those mapped to the 231 variants. A total of 46 genes were replicated in different studies (Supplementary file 1; Tables 2, 3).

### Step 2: *In silico* functional variant annotation and pathway enrichment analysis

#### Variant annotation and functional effect prediction

Variant annotation of the 231 common SNPs showed that chromosome 19 had the highest number of SNPs (*n* = 32), followed by chromosome 17 (*n* = 24 SNPs), and chromosome 11 (*n* = 20 SNPs). The results of the variant annotation are shown in Supplementary file 2; Tables 1, 2. The shared SNPs were mapped to 106 genes. The top 6 genes with the highest number of SNPs were MAP kinase activating death domain (*MADD*) (chromosome (CHR) 11, 9 SNPs); EF-hand calcium binding domain 5 (*EFCAB5*) (CHR 17, 9 SNPs); nectin cell adhesion molecule 2 (*NECTIN2*) (CHR 19, 6 SNPs); cystatin C (*CST3*) (CHR 20, 6 SNPs), apolipoprotein E (*APOE*) (CHR 19, 5 SNPs) and 1-acylglycerol-3-phosphate O-acyltransferase 1 (*AGPAT1*) (CHR 6, 5 SNPs). Variant annotation revealed the association of nine SNPs with drug response (Table 1) and 10 variants were annotated as clinically likely pathogenic/pathogenic (Table 2).

**Table 1:** Summary of T2D-AD shared variants associated with drug response.

CHR	PB	Mapped gene	rs ID	Drug
1	11,796,321	*MTHFR*	rs1801133	l-methylfolate, Vitamin B-complex, Incl. Combinations, methotrexate, bevacizumab, carboplatin, cisplatin, cyanocobalamin, folic acid, pemetrexed capecitabine, fluorouracil, leucovorin, oxaliplatin, clozapine, olanzapine, nitrous oxide
15	78,590,583	*CHRNA5*	rs16969968	nicotine, cocaine, bupropion, Drugs used in nicotine dependence, varenicline, ethanol, Opium alkaloids and derivatives
19	44,905,579	*APOE*	rs405509	Selective serotonin reuptake inhibitor
19	44,908,684	*APOE*	rs429358	acenocoumarol, warfarin, hmg coa reductase inhibitors, Antivirals for treatment of HIV infections, combinations, ritonavir, simvastatin
19	44,908,822	*APOE*	rs7412	atorvastatin, warfarin, Antivirals for treatment of HIV infections, combinations, ritonavir, fenofibrate, fluvastatin, pravastatin
19	44,911,194	*APOE*	rs439401	Warfarin
22	19,963,748	*COMT*	rs4680	nicotine, naloxone, oxycodone, fentanyl, methadone, antipsychotics, opioids, entacapone, propranolol, modafinil, Analgesics, Antiinflammatory agents, non-steroids, Ergot alkaloids, sumatriptan, clozapine, venlafaxine, buprenorphine, fluvoxamine, remifentanil, risperidone
4	88,131,171	*ABCG2*	rs2231142	rosuvastatin, cyclophosphamide, doxorubicin, fluorouracil, imatinib, gemcitabine, dolutegravir, simvastatin, tenofovir, sunitinib, methotrexate, atorvastatin, apixaban, efavirenz, sulfasalazine, fluvastatin, lamotrigine, allopurinol, Opioid anesthetics, Other general anesthetics, volatile anesthetics, gefitinib, capecitabine, fluorouracil, leucovorin, oxaliplatin
6	31,575,254	*LTA-TNF*	rs1800629	etanercept, carbamazepine, sorafenib, carboplatin, gemcitabine, ethambutol, isoniazid, pyrazinamide, rifampin, cyclosporine, mycophenolate mofetil, Tumor necrosis factor alpha (TNF-alpha) inhibitors, atorvastatin

**Table 2:** Summary of T2D-AD common variants clinically likely pathogenic.

CHR	Position	Mapped gene	rs ID	Phenotype associated in ClinVar
1	11,796,321	*MTHFR*	rs1801133	Homocystinuria Due To Methylene Tetrahydrofolate Reductase Deficiency
17	7,676,040	*TP53*	rs11540654	Li-Fraumeni Syndrome, Hereditary Cancer-Predisposing Syndrome
19	44,908,684	*APOE*	rs429358	Familial Type 3 Hyperlipoproteinemia, APOE3 ISOFORM
19	44,908,822	*APOE*	rs7412	Familial Type 3 Hyperlipoproteinemia, Hyperlipoproteinemia Due To APOE1
20	23,637,790	*CST3*	rs1064039	Macular Degeneration, Age-Related
3	39,265,671	*CX3CR1*	rs3732378	Coronary Artery Disease, Resistance To, Human Immunodeficiency Virus Type 1, Rapid Progression To AIDS, Age Related Macular Degeneration 12
6	26,090,951	*HFE*	rs1799945	Hereditary Hemochromatosis, Alzheimer disease Type 1 Familial Porphyria Cutanea Tarda Variegate Porphyria Hemochromatosis Type 1 Microvascular Complications Of Diabetes, Transferrin Serum Level Quantitative Trait Locus 2, Cardiomyopathy, Abnormality Of Iron Homeostasis, Variegate Porphyria
6	26,092,913	*HFE*	rs1800562	Hereditary Hemochromatosis Type 1, Hereditary Cancer-Predisposing Syndrome, Abdominal Pain, Peripheral Neuropathy, Pain, Abnormal Peripheral Nervous System Morphology, Abnormality Of The Male Genitalia, Behavioral Abnormality, Abnormality Of The Nervous System, Cardiomyopathy, Hemochromatosis Type 2, HFE-Related Disorder, Hemochromatosis, Juvenile, Digenic, Alzheimer disease
7	150,999,023	*NOS3*	rs1799983	Susceptibility To Metabolic Syndrome

A total of 66 variants located in 52 genes were identified as missense or stop-gain mutations. Among these, 24 SNPs were predicted to be possibly damaging by PolyPhen2. We identified six SNPs (rs7412, rs2070600, rs4762, rs11540654, rs1799969, and rs751141) located in the *APOE*, advanced glycosylation end-product specific (*AGER*), angiotensinogen (*AGT*), tumor protein p53 (*TP53*), intercellular adhesion molecule 1 (*ICAM1*), and epoxide hydrolase 2 (*EPHX2*) gene, that had the highest pathogenicity score (PolyPhen2 score = 1). Polyphen2 results are provided in Table 3 of Supplementary file 2.

Most SNPs extracted from the included studies were located in the non-coding region and have a “modifier” impact. Please correct the following sentence Functional annotation using the RegulomeDB identified three variants (rs1544210, rs12679834, and rs515071) located in the hematopoietically expressed homeobox *(HHEX)*, lipoprotein lipase *(LPL)*, and ankyrin 1 *(ANK1)* gene which were the most likely regulatory SNPs (Table 3). The full list of RegulomeDB outputs is provided in Table 4 of Supplementary file 2.

**Table 3:** Regulatory T2D-AD shared SNPs.

Gene	rsID	RegulomeDB rank	GTEx e-QTL in brain tissues	TFB motifs	Histone modifications
*HHEX*	rs1544210	1b	*EIF2S2P3* in Substantia nigra (value of *p* = 9.4e^−7^) and Hypothalamus (value of *p* = 0.000025)	USF1, Myc, USF2, DMRT3	
*LPL*	rs12679834	1b		ZNF354C, NR2C2, NKX2-8	Located in Substantia Nigra region marked by H3K4me1 and H3K27ac histone modifications
*ANK1*	rs515071	1b	*ANK1* in Cerebellum (value of *p* = 1.9e^−54^) and in Cerebellum Hemisphere (value of *p* = 7.5e^−49^)	Tcf3	Located in Inferior Temporal Lobe, Angular Gyrus and Anterior Caudate regions marked by H3K27ac histone modifications

Expression quantitative trait loci (e-QTL), Genotype Tissue Expression (GTEx), Transcription factor binding motif (TFB motifs).

To better assess the role of these variants in genetic cis-regulation in the brain, we explored the GTEx pathway. We found an association between the minor allele A rs1544210 and the under-expression of the *EIF2S2P3* pseudogene in the substantia nigra and hypothalamus. Similarly, the minor allele G of the variant rs515071 was associated with decreased *ANK1* expression in the cerebellum and the cerebellar hemisphere. The search for rs12679834 revealed no ci-regulation of this SNP in the brain tissues (Table 3).

**Table 5:** Molecular pathways enriched by shared genes between T2D and AD.

Category	Term ID	Description	Adjusted_*p*_value	Genes
KEGG	KEGG:04979	Cholesterol metabolism	0.0025837551982072883	*LPL, ABCA1, APOC1, APOE*
REAC	REAC:R-HSA-8963898	Plasma lipoprotein assembly	0.0072601471697531315	*ABCA1, APOC1, APOE*
WP	WP:WP430	Statin inhibition of cholesterol production	0.0010409155290130897	*LPL, ABCA1, APOC1, APOE*

Alzheimer’s disease (AD), Kyoto Encyclopedia of Genes and Genomes (KEEG), Reactome (REAC), Type 2 Diabetes (T2D), Wikipathway (WP).

To further explore the regulatory effects of these variants, the FeatSNP database was searched to evaluate the possible epigenetic effects in different brain regions. We identified four potential transcription factor (TF)-binding motifs associated with allele A of rs1544210: USF1, Myc, USF2, and DMRT3. However, histone modification signals associated with the selected SNP were not detected.

A search of the FeatSNP database showed one potential TF-binding motif (NR2C2) associated with the A allele of rs12679834. Our results showed a strong correlation between NR2C2 (nuclear receptor subfamily 2 group C member 2) expression and *LPL* expression in two different brain regions: putamen (*r* = 0.736) and caudate (*r* = 0.651). Furthermore, we found that the region tagged with SNP rs12679834 was enriched for strong active histone modification signals, including H3K4me1 and H3K27ac, in all three brain tissues.

Finally, the results for rs515071 showed that one TF-binding motif (TCF3) was associated with the G allele of this variant. The region tagged by this SNP was enriched for the active histone modification H3K27ac in the inferior temporal lobe, angular gyrus, and anterior caudate region.

**Figure 3:**
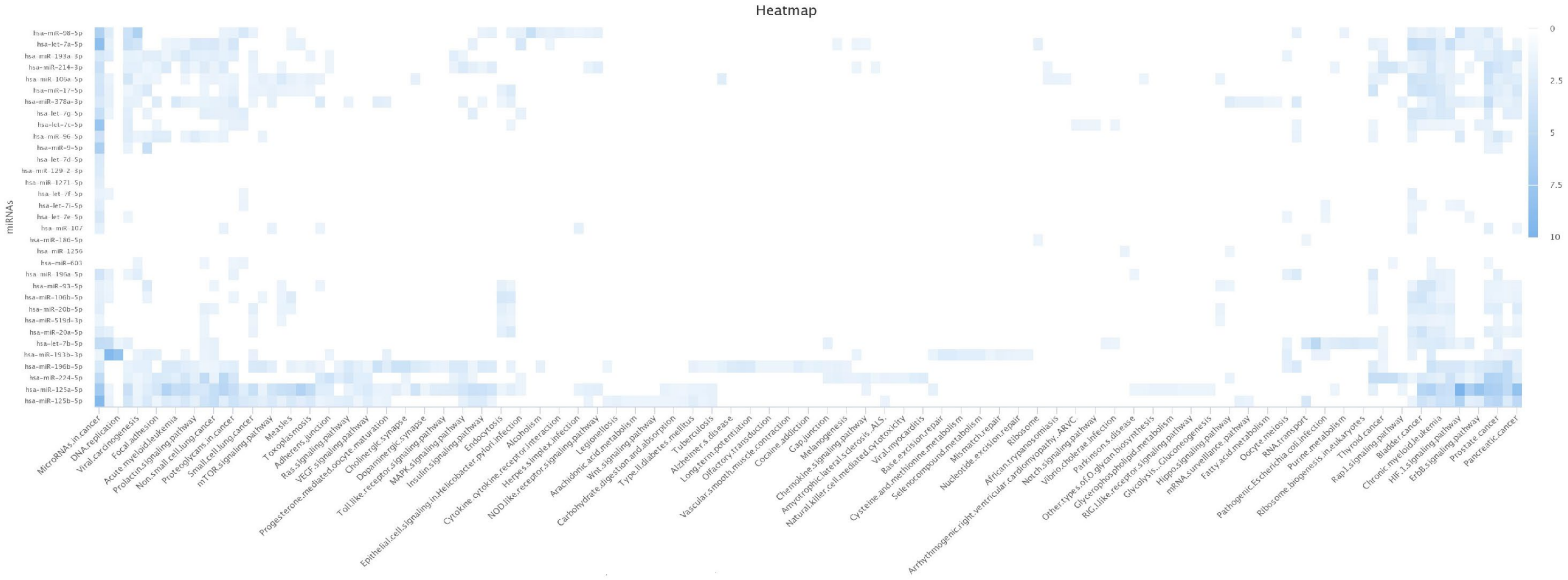
Heatmap of T2D_AD miRNAs enriched pathways. Our results showed that the majority of miRNAs were mapped to several pathways involved in different diseases such as cancer, T2D, AD and insulin signaling pathways. Type 2 Diabetes (T2D); Alzheimer’s disease (AD),micro Ribonucleic Acid (miRNA).

The SNPinfo results showed that only seven variants were predicted to have a potential effect on miRNA-binding sites (Table 4). All the identified SNPs, except one (rs6997), affected miRNAs such as rs6859 that affect hsa-miR-378 (Supplementary file 2; Tables 5, 6). Our results showed that the majority of miRNAs were mapped to several pathways involved in different diseases such as cancers,T2D, AD, and insulin signaling pathways (Figure 3).

**Table 4:** Summary of variants with potential effect on miRNAs binding sites.

Position	Mapped gene	SNP	miRNA	Disease
1: 109275684	*CELSR2*	rs629301	hsa-miR-338-3p, hsa-miR-224, hsa-miR-214, hsa-miR-186, hsa-miR-193b, hsa-miR-193a-3p, hsa-miR-193, hsa-miR-103, hsa-miR-107, hsa-miR-485-5p, hsa-miR-9, hsa-miR-125b, hsa-miR-125a-5p, hsa-miR-125a, hsa-miR-431, hsa-miR-17-5p, hsa-miR-106a, hsa-miR-20a, hsa-miR-106b, hsa-miR-93, hsa-miR-519d, hsa-miR-20b, hsa-miR-17, hsa-miR372, hsa-miR-20, hsa-miR-1,271, hsa-miR-96	
19: 44878777	*PVRL2*	rs6859	hsa-miR-378	Late onset Alzheimer’s Disease
3: 49357401	*GPX1*	rs1050450	hsa-miR-1,233, hsa-miR-129-3p	Breast cancer, Lung cancer, Kashin-Beck disease
3: 9757089	*OGG1*	rs1052133	hsa-miR-1,256	Lung cancer, Colorectal cancer, Gallbladder cancer
6: 32180626	*RNF5*	rs8365	has-miR-196a, has-miR-196b, has-let-7b, has-let-7d, has-let-7i, has-let-7a, has-let-7f, has-let-7c, has-let-7e, has-let-7 g, has-miR-98	
7: 75986787	*POR*	rs17685	hsa-miR-603	

#### Pathway enrichment analysis and visualization

**Figure 4:**
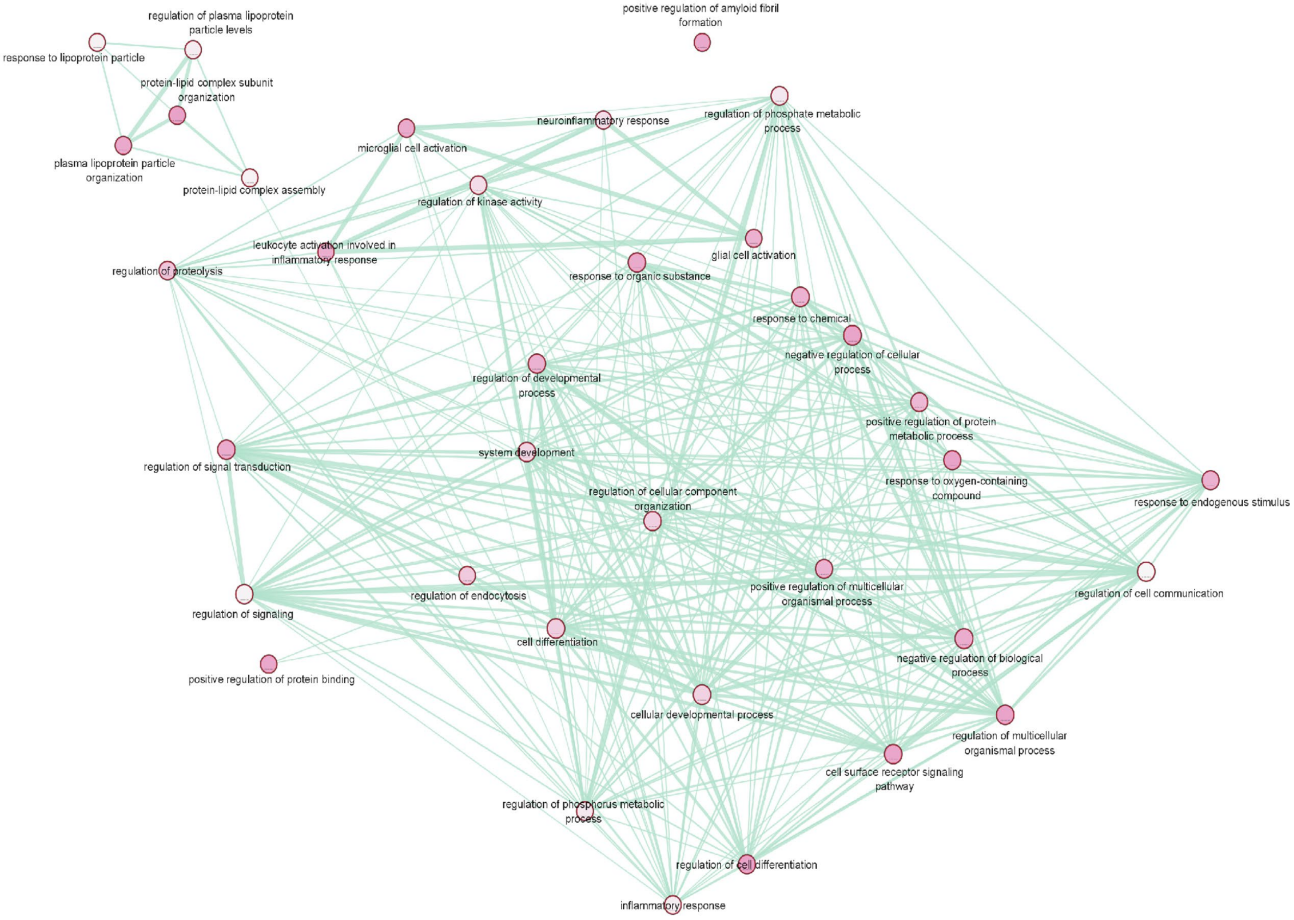
Pathway enrichment analysis of T2D and AD common genes. The main enriched pathways were: lipid subunit organization, positive regulation of protein binding, positive regulation of amyloid fibril formation, microglial cell activation (*p*-value = 0.01).

To gain a deeper understanding of T2D and AD common genes we conducted a pathway enrichment analysis using g:Profiler. In order to obtain accurate results in terms of the relationship between these diseases, the gene set was limited to genes replicated among the selected studies. The g:Profiler pathway enrichment analysis results are shown in Table 5 and Supplementary files 3 and 4. The obtained results were then visualized using EnrichmentMap (Figure 4). The main enriched pathways were: lipid subunit organization, positive regulation of protein binding, positive regulation of amyloid fibril formation, microglial cell activation, (value of *p* =0.01). Furthermore, analysis of the KEGG pathway Reactome, and WikiPathway revealed enrichment of cholesterol metabolism (*p*-value = 2.584 × 10^−3^) plasma lipoprotein assembly (*p*-value = 7.260 × 10^−3^), and Statin inhibition of cholesterol production (*p*-value = 1.041 × 10^−3^).

### Step 3: Genetic landscape analysis of shared T2D-AD genes

#### Statistical analysis

**Figure 5:**
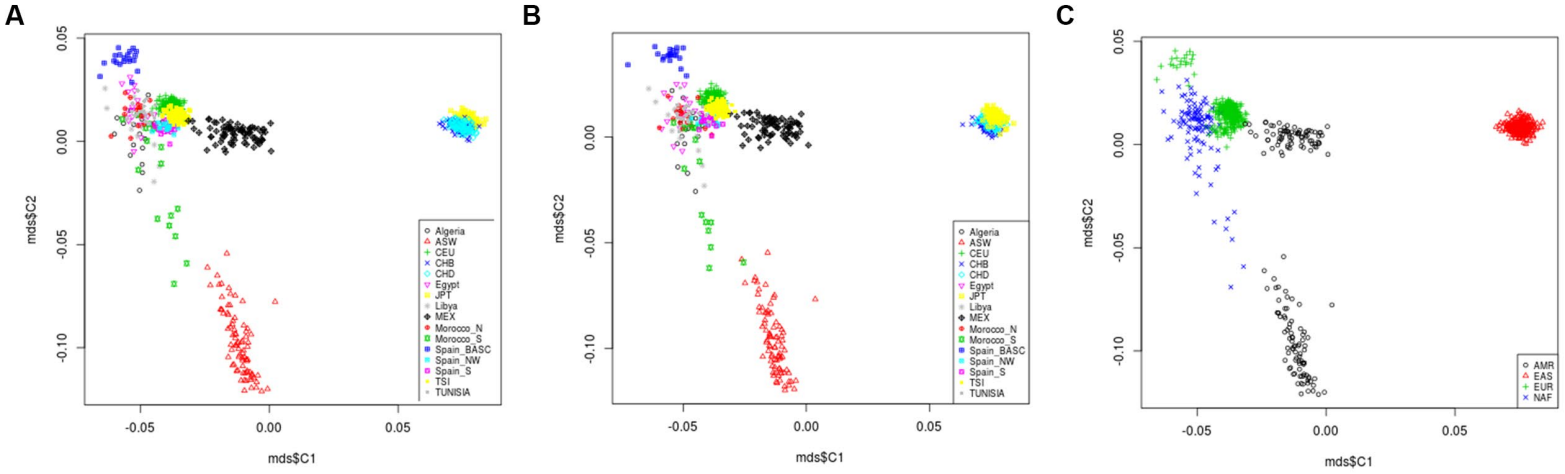
Multidimensional scaling plot of T2D and AD shared variants landscape in worldwide populations. The plot shows that North African populations (Algeria, Egypt, Libya, Morocco-N, Morocco-S, Tunisia) are clustered within the European populations (CEU, Sapin-S, Spain-Basic, Spain-NW, and TSI) and distinguished from the American (ASW, MEX) and Asian (CHB, CHD, JPT) populations **(A,B)**. Better individualization was observed in MDS performed across continents. In addition, there is a great divergence among the North African (NAF), American (AMR) and East Asian (EAS) groups. However, slight proximity was observed between the NAF and European EUR clusters **(C)**. *Rare variants are those with MAF < 10^−2^. **Comment variants are those with MAF > 10^−2^. Type 2 Diabetes (T2D); Alzheimer’s disease (AD).

All variants located in in T2D-AD common genes were extracted from the genotyping data of 829 individuals from the studied populations (Supplementary file 5). A total of 212,688 variants were identified after merging of the genotyping data. Among them, we did not find the rare variants of interest reported in the literature. Then, we generated a second set of common variants after excluding SNPs with MAF < 10^−2^. In total, 123,115 common variants were retrained. MDS analysis describing the genetic landscape of these genetic variants was generated for the two sets of variants (set 1 with MAF < 10^−2^ = 212,688 variants, and set 2 without MAF < 10^−2^ = 123,115). There was no difference between the MDS plots generated by the two sets of variants. The MDS plot showed that the North African populations (Algeria, Egypt, Libya, Morocco-N, Morocco-S, Tunisia) were clustered within the European populations (CEU, Spain-S, Spain-Basic, Spain-NW, and TSI) and distinguished from the American (ASW, MEX) and Asian (CHB, CHD, JPT) populations (Figures 5A,B). Better individualization was observed in MDS performed across continents. In addition, there is great divergence among the North African (NAF), American (AMR), and East Asian (EAS) groups. However, slight proximity was found between the NAF and EUR clusters (Figure 5C).

Among the 231 variants of interest, only 49 risk alleles variants were identified in the studied populations. Interethnic comparison based on the selected MAF variants revealed significant differences at the level of 11 SNPs between North African, European, and East Asian populations. The 11 SNPs were located in *DNM3, CFH, PPARG, ROHA, RAGE, CLU, BDNF1, CST9*, and *PLCG1* genes (Supplementary file 6). No significant differences were found for the risk allele frequency of candidate genes between the North African and American populations (Supplementary file 7).

#### Analyses of population genetic structures

**Figure 6:**
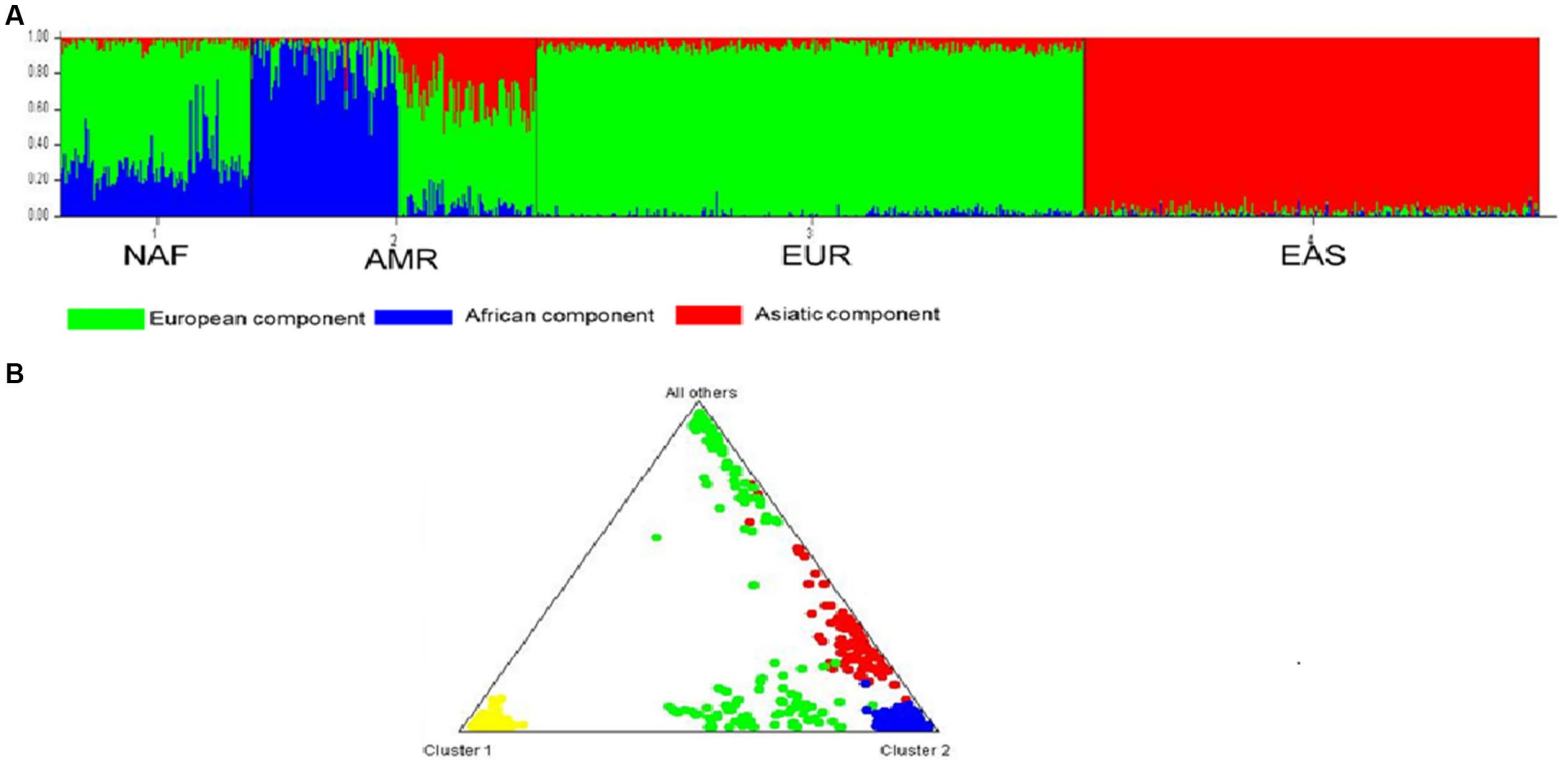
STRUCTURE analysis of the genetic relationship between the three group of populations. **(A)** The Bar plot shows three components: Africa, Asia and Europe. STRUCTURE analysis confirmed the ancestral diversity of the North African populations with evidence of the predominance of European component. **(B)** The Triangle of the structure shows that the NAF cluster is close to the EUR cluster and distinct from the EAS cluster. North African (NAF), European (EUR), and East Asian (EAS).

To determine the distribution of common T2D-AD variants between the studied populations, we adopted a Bayesian iterative algorithm using the STRUCTURE software. In accordance with Evanno’s ΔK method for STRUCTURE, the hypothetical K number of ancestries was set at three (K = 3) to detect the most likely number of genetic clusters (Supplementary file 8). The Bar plot shows three components: Africa, Asia, and Europe. STRUCTURE analysis confirmed the ancestral diversity of the North African populations with evidence of the predominance of European components (Figure 6A). The Triangle of the structure shows that the NAF cluster is close to the European cluster and distinct from the EAS cluster (Figure 6B).

## Discussion

In the present study, we collected T2D-AD common variants and genes from the literature. Then, we analyzed their functional predictions and pathways. Finally, we explored the genetic variability of the collected variants among North African populations in comparison with other populations worldwide.

### Common variants and genes between T2D and AD

Our literature search revealed 231 variants and 363 genes in common between T2D and AD. Annotation of the 231 shared SNPs showed that *MADD* and *EFCAB5* harbored the highest number of these variants (nine SNPs). *MADD* gene, also known as *IG20*, plays a critical role in the development of glucose intolerance (Dupuis et al., 2010; Hu et al., 2010; Strawbridge et al., 2011; Wagner et al., 2011) and AD (Del Villar and Miller, 2004; Hassan et al., 2021). In accordance with our findings, a large-scale genome-wide cross-trait study identified *MADD* as the only gene significantly associated with AD and fasting glucose exclusively in pituitary tissue; it is also the only shared gene found in both cross-trait Meta-analysis and Transcriptomic-wide association studies. Thus, the pituitary gland may link T2D and AD by regulating glucose metabolism and neuronal viability through *MADD* (Zhu et al., 2019).

Regarding the *EFCAB5* gene, it encodes the EF-hand calcium-binding domain 5. Our results are in accordance with those of Karki et al. (2020) highlighting the importance of this gene inT2D and AD development. Among the nine identified variants in this gene, two SNPs (rs9902453 and rs7221743) are associated with coffee consumption (Cheung et al., 2012; Cornelis et al., 2015). In this context functional studies showed the protective role of coffee consumption against AD (Kwok et al., 2016; Zhou et al., 2018) and T2D-associated memory impairment through adenosine A2 receptor (ADORA2A) blockage (Duarte et al., 2019). Thus, we suggest that variants in *EFCAB5* could affect memory impairment in T2D subjects.

We found six SNPs located in *NECTIN2*, which encodes the nectin cell adhesion molecule 2 protein involved in T-cell signaling (Zhu et al., 2016). These variants have been previously reported to be shared between T2D and AD (Wang et al., 2017). Indeed, these variants are associated with lipid metabolite measurements, emphasizing their critical role in the development of AD in T2D patients (Xiao et al., 2022).

*CST3* encodes cystatin C inhibitors of cysteine proteases (Maniwa et al., 2020). It is one of the genes harboring the highest number of common SNPs, between T2D and AD (Karki et al., 2020). A recent study showed that CST3 protein aggregation abolishes its function and slightly increases amyloid-beta 1–40 (Aβ1-40) fibril formation, enhancing neurodegeneration (Sheikh et al., 2021). Furthermore, the exogenous Cystatin C induces impairment of insulin signaling in hippocampal neurons, which could promote cognitive decline and AD development (Luo et al., 2018). In contrast, other studies have suggested that Cystatin C exerts neuroprotective effects by inhibiting cysteine proteases, rescuing neurodegeneration, inhibiting Aβ oligomerization and amyloid fibril formation, inducing autophagy, and neurogenesis (Mathews and Levy, 2016). This discrepancy may be explained by Cystatin C conformation or its levels in the brain. Indeed, the shared T2D-AD variant (rs1064039) was previously associated with reduced Cystatin C levels owing to impaired signal peptide cleavage (Benussi et al., 2003). We hypothesized that *CST3* plays a crucial role in the development of T2D-induced AD pathology through the regulation of cerebral amyloid angiopathy and insulin signaling in a dose dependent manner. Further studies are needed to determine its exact function in T2D-inducing AD condition.

We found five shared SNPs between T2D and AD within the *APOE* gene. These findings are in line with the literature. *APOE* is a leading factor for AD development in T2D subjects (Zhen et al., 2018; Shinohara et al., 2020). Three variants among the five were associated with body mass index (Yengo et al., 2018). Our findings support the synergic effect of obesity and *APOE* genotype on the development of T2D and AD (Jones and Rebeck, 2018).

*AGPAT1* encodes for 1-acylglycerol-3-phosphate O-acyltransferase 1. It harbors five SNPs among the 231 T2D-AD shared variants identified in this study. Deletion of this gene induces low glucose and lipid plasma levels, as well as neurological disturbances (Agarwal et al., 2017). These findings emphasize the importance of *AGPAT1* in the regulation of glucose homeostasis and neuron viability. Further studies are needed to investigate its role in T2D and AD.

The functional annotation of the coding variants revealed six probably damaging SNPs located in *APOE, AGER*, *AGT*, *TP53*, *ICAM1*, and *EPHX2* gene. All the variants have been identified by the Karki et al. (2020), study. Interestingly the two variants rs7412 and rs2070600 have been reported by other studies (Wang et al., 2016; Kim et al., 2022). Minor allele (T) carriers of the variant rs7412 are classified as APOEε2 carriers. It has been largely proven that *APOEε2* has a protective effect against AD (Shinohara et al., 2016). Controversially, Shinohara et al., (2020) showed that *APOEε2* accelerates cognitive decline in diabetic patients by 4 years. This could be explained by the synergic effect between diabetes (Hardigan et al., 2016; Peng et al., 2021) and the *APOEε2* genotype in enhancing neurovascular impairment and tauopathies (Kim et al., 2022).

The second SNP (rs2070600) located in *AGER* (advanced glycosylation end-product specific receptor), causes a conversion at position 82 from glycine to serine (G82S) responsible for a decrease of AGER proteolyze and increase of AGEs plasmatic levels (Serveaux-Dancer et al., 2019). Several studies shed the light on the role of AGEs in T2D and AD through oxidative stress and amyloid regulation mechanisms (Michailidis et al., 2022). As a result, we hypothesize that T2D patient carriers of these risk allele variants have a greater risk to develop AD.Likely, the functional annotation of non-coding variants revealed three top-ranked SNPs rs1544210, rs12679834, and rs515071 located, respectively, in *HHEX, LPL*, and *ANK1* gene. The minor allele A of the variant rs1544210 is linked to an under-expression of *EIF2S2P3* pseudogene in the substantia nigra and hypothalamus regions. *EIF2SS2P3* is a pseudogene located in chromosome 10. Transcriptomic-wide association studies revealed a significant enrichment of *EIF2S2P3* for depressive symptoms, T2D (Génin, 2020) and T2D patients skipping breakfast (Chen et al., 2022), but its function remains unclear. Available evidence showed an increased risk for dementia development in T2D patients with depression (Katon et al., 2012). These findings highlights the role of *EIF2S2P3* pseudogene in the development of T2D-induced dementia through enhancing depression. Further studies should be conducted to assess the role of *EIF2S2P3* in T2D and AD development.

The same allele was associated with TFB motif “USF1,” that regulates *APOE* gene a major linking factor between T2D and AD (Isotalo et al., 2012).

The second top-ranked SNP is rs12679834 located in the *LPL* gene. We found a TBF motif (NR2C2) with a high score associated with the G allele of this variant. NR2C2, also known as TR4, is an orphan nuclear receptor targeting many genes involved in metabolism including *APOE* (Fogarty et al., 2013). This variant was also marked by H3K4me1 and H3K27ac histone modification in three brain regions. Furthermore, an increased expression of *LPL* in microglia appears to have a protective effect against AD (Keren-Shaul et al., 2017) and obesity (Gao et al., 2017). We suggest that rs12679834 possesses a protective effect against T2D and AD development via increasing lipid and lipoprotein uptake in the Central nervous system (CNS).

The third SNP was rs515071 located in the *ANK1* gene. The minor allele G of this variant was associated with decreased expression of *ANK1* gene in the cerebellum and cerebellar hemisphere regions. In agreement with our results, the GG genotype of the rs515071 variant is associated with a greater risk for T2D (Sun et al., 2017, p. 1) and AD (Chi et al., 2016). A reduced expression of *ANK1* gene could enhance T2D and AD development by affecting mediated metabolism, signal transduction (Sun et al., 2017), and inflammatory process (Morris et al., 2019).

### Shared miRNA and pathways between T2D and AD

Six SNPs reported in our study were found to affect miRNA molecules implicated in LOAD, insulin signaling, and T2D pathways. miR-1965-5p was enriched in the AD, T2D, and insulin signaling pathways. It has a positive effect on insulin biogenesis by enhancing insulin activity (Panda et al., 2014). miR-196b-5p down regulation has been implicated in innate immune response, apoptosis, and depression (Zhang et al., 2018). Inconsistent results have been found to be associated with the regulation trend in patients with AD (Pichler et al., 2017). We suggest that miR-196b-5p may play a protective role against T2D and AD development through insulin, immune response, and apoptosis regulation. However, further studies are required to elucidate their role in T2D and AD. Controversially, miR-378, previously associated with LOAD (Lusardi et al., 2017), was also enriched in the insulin-signaling pathway. Interestingly, miR-378 induces insulin resistance by targeting *P110a* and *SIRT7* (Deng and Guo, 2019). Furthermore, genetic depletion of miR-378a-3p ameliorates inflammatory stress and insulin resistance via protein kinase R inhibition (Wang et al., 2021). Likewise, upregulation of this miRNA has also been found in patients with AD (Dong et al., 2021; Li and Cai, 2021). This evidence emphasizes its role in T2D-induced AD through Central insulin signaling impairment (Gabbouj et al., 2019).

A previous study showed that miR-125a-5p and miR-125b-5p shared between T2D and the insulin pathway could ameliorate gluconeogenesis, glycogen synthesis (Xu et al., 2018), and insulin sensitivity (Yu et al., 2019). Interestingly, the same miRNA was found to be downregulated in the gray matter of patients with AD (Wang et al., 2011). We found that miR-98-5p was enriched in the insulin-signaling pathway. Decreased expression of this miRNA has been observed in T2D patients (Kokkinopoulou et al., 2019). The same study also reported a negative correlation between miR-98-5p and insulin levels in patients. Interestingly, Chen et al. (2019) found that in AD mice, miR-98 binds to HEY2 inducing a decrease of Aβ production, improve oxidative stress, and mitochondrial dysfunction through activating the Notch signaling pathway. We hypothesized that low levels of miR-98-5p could serve as a biomarker for insulin resistance and Aβ aggregation. A recent study found a significant downregulation of miR-214-3p blood levels in T2D patients (Avgeris et al., 2020). Similarly, miR-124-3p is downregulated in patients with AD and animal models (Kou et al., 2020), suggesting its potential role as a biomarker and therapeutic target for insulin resistance (Cheng et al., 2020) and cognitive defects (Zhang et al., 2018).

For a more in-depth understanding of the shared genes, we conducted a pathway enrichment analysis. The results revealed that the replicated genes were mainly enriched in lipid subunit organization, positive regulation of protein binding, positive regulation of amyloid fibril formation, microglial cell activation, cholesterol metabolism, plasma lipoprotein assembly, and Statin inhibition of cholesterol production pathways. Our findings are supported by those of previous studies. Plasma protein binding (PPB) has been implicated in several mechanisms, particularly drug binding and pharmacokinetics (Smith et al., 2010). A recent study identified an enrichment of positive regulation of PPB in 3 × Tg-AD mice fed rosmarinic acid, a preventive molecule against AD (Yamamoto et al., 2021). Rosmarinic acid is a potent suppressor of Aβ and an inhibitor of phosphorylated tau accumulation (Yamamoto et al., 2021). Interestingly, Rosmarinic acid possesses a therapeutic effect against T2D through the remodeling of amyloid aggregates (Wu et al., 2021). We identified, for the first time, the implication of positive regulation of PPB in T2D and AD. We hypothesized that the regulation of the PPB pathway could serve as a potential therapeutic target for these diseases.

The accumulation of amyloid fibrils is a hallmark of several degenerative diseases including T2D and AD. Insulin resistance promotes the oxidative stress generation and proinflammatory cytokines secretion in beta-cells inducing mitochondrial dysfunction and accumulation of protein aggregates, including human islet amyloid polypeptide (hIAPP) (Rocha et al., 2020). The latter can across the blood–brain barrier (BBB) inducing AD pathology (Lupaescu et al., 2022; Marrano et al., 2023).

Furthermore, amyloid deposition causes microglial and astrocyte activation leading to cytotoxic molecules release (Lupaescu et al., 2022). Recent study has demonstrated that hyperinsulinemia impaired GLUT4 translocation inducing mitochondrial fission, microglial M1 polarization, and neuroinflammation (Yang et al., 2022). Moreover, long-term high fat diet induces microglial M1 polarization which explains obesity/diabetes-associated cognitive impairment (Wu et al., 2020).

Cholesterol metabolism involves energy metabolism, cell membrane composition, and myelination. Dysregulation of these biological processes induces several pathologies, mainly T2D and AD. Reports suggested that a long-term high-fat diet could induce AD by enhancing Aβ and phosphorylated tau accumulation (Czuba et al., 2017). Downregulation of cholesterol biogenesis has been observed in diabetic (Suzuki et al., 2010) and AD (Varma et al., 2021) brains. Cholesterol is biosynthesized in astrocytes via the Bloch pathway and is transported to neurons by APOE via the ABC transporter. For utilization by neurons, APOE-containing cholesterol should be absorbed by LRP1/LDLR (Czuba et al., 2017). A previous study successfully demonstrated that insulin resistance suppresses *LRP1* expression, which may further compromise insulin signaling and cholesterol metabolism in neurons (Liu et al., 2015). Thus, our pathway enrichment analysis confirmed previous findings supporting the role of cholesterol metabolism and lipoprotein processes as linking factors between T2D and AD.

### T2D-AD genetic landscape in North African populations

It is likely that genetic background plays an important role in the development of preventive strategies targeting modifier risk factors, such as T2D. Despite the high prevalence of T2D (Magliano et al., 2021) and AD (Nichols et al., 2022) in North African populations, we noticed an under or non-representation of these groups in the consortiums investigating these diseases (Martin et al., 2019). Taking all these evidences into consideration, it is important to dissect the genetic landscape of T2D-AD shared genes in North Africa in comparison with other well-studied populations. We determined the genetic landscape of T2D-AD shared genes in 829 individuals from 16 different populations whose genotyping data are publicly available [African ancestry in the South Western USA(ASW), Mexican ancestry living in Los Angeles, California, USA (MEX), Western European ancestry populations of Utah from the CEPH collection (CEU), Toscani people of Italy (TSI),South Spain (Spain_S), North Spain (Spain_N), North West of Spain (Spain_NW), Spain Basic populations (Spain_BASC), Han Chinese in Beijing, China (CHB),Chinese population of metropolitan Denver, Colorado, USA (CHD), Japanese in Tokyo, Japan; (JPT), Individuals from North Africa: Algeria (Algeria), Egypt (Egypt), Libya (Libya), Tunisia Douiret (TN_Ber), South Morocco (Morocco_S), and North Morocco (Morocco_N),],. MDS analysis showed genetic similarity among North African populations (Algeria, Egypt, TN_Ber, Morocco_N, Morocco_Sand Libya), reflected by a consistent cluster. A better individualization of the North African populations was identified when the MDS analysis was conducted at the population group level. A slight similarity between North African and Southwestern European populations (CEU, Spain-S, Spain_NW, and TSI) was detected. However, a great divergence between North African and East Asian populations was observed in the two MDS representations. These results were further confirmed by STRUCTURE analysis conducted on four clusters of populations: North African, European, East Asian, and American. STRUCTURE representation shows a high admixture of the genetic structure of North African populations consisting mainly of European and African components, with minimum penetrance of East Asian components. Our findings are consistent with those of previous studies. Indeed, several genes/polymorphisms in T2D (Chande et al., 2020) and AD (Rubin et al., 2021) are highly variable among ethnic groups. Similar genetic positioning was observed among North African, European, and Asian populations regarding Metabolic Syndrome (MetS) pharmacogenes (Jmel et al., 2018). It is important to note that genes explored by Jmel et al. (2018) were also investigated in our study because MetS share several mechanisms with T2D and AD (Hayden, 2019). The genetic positioning of the North African cluster could be explained by the high ethnic heterogeneity of these populations. North Africans are multi-ethnic populations with several ancestral components: Middle Eastern, Sub-Saharan African, European, and autochthonous (Arauna et al., 2019). The high heterogeneity of the T2D-AD genetic background in North African populations reflects previous historical events such as invasion and migration (Botigué et al., 2013; Arauna et al., 2017; Fregel et al., 2018). Our results also support the conserved and ancient divergence between the North African and East Asian populations going back 550 centuries ago (Tateno et al., 2014). Thus, the non-replication of some genetic biomarkers of T2D (Baroudi et al., 2009; BaroudiOuederni et al., 2009; Ezzidi et al., 2009; Turki et al., 2012) and AD (Smach et al., 2011, p. 1; Rassas et al., 2013; Landoulsi et al., 2018) in the North African group could be due to its high heterogeneity and unicity. Indeed, among 231 risk variants studied, only 49 SNPs were present in the North African group. This could be the result of ethnic selection because some AD variants are also ethnicity-specific biomarkers (Huang et al., 2017) or due to the limited size of the North African populations investigated.

Furthermore, the inter-ethnic risk allele frequency comparison of the 49 variants between North African populations and other population groups revealed significant differences in allele frequency of 11 SNPs between North African, European, and East Asian populations located in *DNM3*, *CFH*, *PPARG*, *ROHA*, *AGER*, *CLU*, *BDNF1*, *CST9*, and *PLCG1* genes.

MAF of two variants, rs4504922 and rs7539972, located in the dynamin 3 (*DNM3*) gene, was significantly different between North African and European populations and between North African and East Asian populations. *DNM3* is enriched in the Fc gamma R-mediated phagocytosis pathway associated with AD and T2D (Hao et al., 2015; Caputo et al., 2020). These variants were previously identified as SNPs shared between T2D and AD (Caputo et al., 2020). We suggest that North African carriers of these risk allele variants may be at increased risk of T2D and AD.

The rs800292 G > A SNP, located in the complement factor H (*CFH*) gene, has been previously reported to be associated with a higher risk of age-related macular degeneration (Guindo-Martínez et al., 2021) and diabetic retinopathy (Wang et al., 2013).

Furthermore, the inter-ethnic comparison of risk alleles revealed a significant difference in the MAF of three variants (rs6809832, rs6997, and rs11715915) located in peroxisome proliferator activated receptor gamma (*PPARG*) and Ras homolog family member A (*RHOA*) genes between North African and East Asian populations. These variants have been associated with increased BMI and HbA1c levels (Merino et al., 2017; Yengo et al., 2018; Barton et al., 2021; Jurgens et al., 2023). We hypothesized that North African carriers of risk alleles of these variants may have an increased risk of developing obesity and insulin resistance pathologies, such as T2D and AD (Magliano et al., 2021; Nichols et al., 2022).

Moreover, we identified significant differences in the MAF of three SNPs (rs2070600, rs11136000, and rs6265) located in *AGER*, clusterin (*CLU*), and brain derived neurotrophic factor (*BDNF1*) between North African and East Asian populations. These variants have been previously associated with MCI/AD development in T2D subjects (Cai et al., 2016; Wang et al., 2016; Daily and Park, 2017; Stepler and Robinson, 2019; Bradley, 2020). The high MAF of these variants in North Africans could partly explain their greater risk of developing T2D-AD pathology, (Magliano et al., 2021; Alzheimer’s Disease Facts and Figures, n.d.).

The variant rs3004145 C > G, located downstream of the Cystatin C9 (*CST9*) gene, presents a significant MAF difference between North African and East Asian populations. This variant has previously been associated with elevated cystatin C levels in the European population (Jurgens et al., 2023). High serum cystatin C levels have been previously associated with an increased risk of T2D (Yuan et al., 2022), T2D-related neuropathy (Hu et al., 2014), and AD (Straface et al., 2005). Thus, we suggest that North African carriers of the rs3004145 G allele may have an increased risk of T2D-induced AD.

Finally, our statistical analysis revealed a significant MAF difference in the exonic variant rs753381 T > C between North African and European populations. This variant is located in the phospholipase C gamma 1 (*PLCG1*) gene, recently identified as a potential therapeutic target for T2D (Ganekal et al., 2023). It has previously been associated with metabolic syndrome (Brown and Walker, 2016) and elevated serum cholesterol, LDL, and ApoB levels in individuals of African, East Asian, European, Hispanic, and South Asian ancestry (Graham et al., 2021). We suggest that the differences in rs753381 C allele frequency in North Africa explain the low plasma levels of TC, LDL-C, and ApoB compared to European populations (Najah et al., 2013). Further studies are required to elucidate the relationship between this variant and T2D-induced AD development in North Africa.

## Study highlights and testable hypotheses

The present study generates several hypotheses: (1) *MADD* and *AGPAT1* genes regulate glucose homeostasis and neuronal viability. (2) *EFCAB5* could be a potential pharmacogene for ADORA2A agonist anti-AD therapies. (3) *NECTIN2* plays a critical role in T2D-induced AD through regulating lipid metabolism. (4) *CST3* regulates cerebral amyloid angiopathy and insulin signaling in a dose dependent manner. (5) Individuals carrier of rs7412, rs1800562, rs2070600 rs1544210, rs12679834, and rs515071 risk alleles are of great risk to develop T2D and AD. (6) miR-378, miR-125a-5p, miR-125b-5p, miR-196b-5p, miR-98-5p, and miR-214-3p are potential therapeutic target for T2D-induced AD. (7) Plasma protein binding pathway could serve as a potential therapeutic target for T2D-induced AD. (8) North African’s carrier of minor alleles of variants located in*, DNM3*, *CFH*, *PPARG*, *ROHA*, *AGER*, *CLU*, *BDNF1*, *CST9*, and *PLCG1* genes are of great risk to develop T2D and AD.

## Study limitations

Although our study’s results give rise to several hypotheses consistent with the published literature, we also have some limitations. First, our search strategy has been limited to one database “PubMed” with one query for search builder. This strategy can lead to information leakage. Therefore, other datasets and search terms should be examined to consolidate our findings. Second, the restricted size of the studied populations, especially in North Africa, could lead to fewer genetic variations present in these populations. Finally, the predicted results should be supported by further experimental studies. Despite these limitations, our study findings are relevant and pave the way to further investigation because of their general consistency with previous results.

## Conclusion

Our study contributes to efforts made to better understand the genetic variability and molecular mechanisms shared between T2D and AD. It is well established that the determination of the genetic component of these diseases could help develop new diagnostic and therapeutic strategies in the context of precision medicine. However, the promise of precision genomic medicine cannot be fulfilled without a broad representation of the global population. Here, we identified pathogenic variants and regulatory pathways shared between these diseases. Our study is the first to investigate the genetic landscape of shared T2D-AD genes in North African populations in comparison to other worldwide populations. Our results support the high heterogeneity and the unicity of North African populations regarding T2D and AD common genes. The inter-ethnic comparison between North African populations and worldwide populations revealed significant difference of eleven risk allele frequency variants. This finding might be one of the contributing factors to the higher prevalence of T2D and AD in North African populations. Furthermore, our results could pave the way for new target gene sequencing or functional follow-up of putative loci to investigate the exact role of these variants in North African populations. Finally, we emphasize the importance of further ethnicity-specific contributions in omics studies for a better understanding of the link between T2D and AD, and for developing an accurate diagnosis using personalized genetic biomarkers.

## Data availability statement

The original contributions presented in the study are included in the article/Supplementary material, further inquiries can be directed to the corresponding author.

## Ethics statement

Ethical review and approval was not required for the study on human participants in accordance with the local legislation and institutional requirements. Written informed consent for participation was not required for this study in accordance with the national legislation and the institutional requirements.

## Author contributions

WB designed the study, collected and curated the original data, conducted analysis and validated results, and drafted the initial manuscript. HJ designed the study, collected and curated the original data, conducted and validated analysis, and wrote—reviewed and edited the manuscript. NK, IG, HD, and MH helped to revise the manuscript. RK conceived the idea, designed the study, supervised the analysis, validated results, wrote—reviewed and edited the manuscript. All authors contributed to the article and approved the submitted version.

## Funding

This work was funded by The Tunisian Ministry of Public Health and the Tunisian Ministry of Higher Education and Scientific Research (The scholarship of Wided Boukhalfa and the salaries of HJ, HD and RK).

## Conflict of interest

The authors declare that the research was conducted in the absence of any commercial or financial relationships that could be construed as a potential conflict of interest.

## Publisher’s note

All claims expressed in this article are solely those of the authors and do not necessarily represent those of their affiliated organizations, or those of the publisher, the editors and the reviewers. Any product that may be evaluated in this article, or claim that may be made by its manufacturer, is not guaranteed or endorsed by the publisher.

## Supplementary material

The Supplementary material for this article can be found online at: https://www.frontiersin.org/articles/10.3389/fnagi.2023.1114810/full#supplementary-material

## Footnotes

^1^ https://pubmed.ncbi.nlm.nih.gov/

^2^ https://regulomedb.org/regulome-search/

^3^ www.internationalgenome.org

^4^ https://www.R-project.org/

## Abbreviations

ABC transporter: ATP Binding Cassette transporter
AD: Alzheimer’s disease
ADORA2A: Adenosine A2a receptor
AGER: Advanced glycosylation end-product specific
AGPAT1: 1-acylglycerol-3-phosphate O-acyltransferase 1
AGT: Angiotensinogen
AMR: American
ANK1: Ankyrin 1
ApoB: Apolipoprotein B
APOE: Apolipoprotein E
ASW: African ancestry in the South Western USA
Aβ: Amyloid-beta
BBB: Blood–brain barrier
BDNF1: Brain derived neurotrophic factor
BMI: Body mass index
CEU: Northwestern and Western European ancestry populations of Utah from the CEPH collection
CFH: Complement factor H
CHB: Han Chinese in Beijing, China
CHD: Chinese population of metropolitan Denver, Colorado, USA
CLU: Clusterin
CNS: Central nervous system
CST3: Cystatin C
CST9: Cystatin C9
DMRT3: Doublesex and mab-3 related transcription factor 3
DNM3: Dynamin 3
EAS: East Asian
EFCAB5: EF-hand calcium binding domain 5
EIF2S2P3: Eukaryotic translation initiation factor 2 subunit 2 beta pseudogene 3
EPHX2: Epoxide hydrolase 2
EUR: European
GLUT4: Solute carrier family 2
H3K27ac: Histone 3 lysine 27 acetylation
H3K4me1: Monomethylation of lysine 4 on histone H3 protein subunit
HbA1c: Glycated hemoglobin
HHEX: Hematopoietically expressed homeobox
hIAPP: Human islet amyloid polypeptide
HWE: Hardy–Weinberg equilibrium
ICAM1: Intercellular adhesion molecule 1
JPT: Japanese in Tokyo, Japan
LD: Linkage disequilibrium
LDL: Low-density lipoprotein
LOAD: Late-onset Alzheimer’s disease
LPL: Lipoprotein lipase
MADD: MAP kinase activating death domain
MAF: Minor allele frequency
MDS: Multidimensional scaling plot
MEX: Mexican ancestry living in Los Angeles, California, USA
miRNA: Micro ribonucleic acid
Morocco_N: North Morocco
Morocco_S: South Morocco
Myc: MYC proto-oncogene, bhlh transcription factor
NAF: North African populations
NECTIN2: Nectin cell adhesion molecule 2
NR2C2: Nuclear receptor subfamily 2 group C member 2
PLCG1: Phospholipase C gamma 1
PPARG: Peroxisome proliferator activated receptor gamma
ROHA: Ras homolog family member A
SNP: Single nucleotide polymorphism
Spain_BASC: Spain Basic populations
Spain_N: North Spain
Spain_NW: North West of Spain
Spain_S: South Spain
T2D: Type 2 diabetes
TC: Total cholesterol
TCF3: Transcription factor 3
TFB: Transcription factor binding motif
TN_Ber: Tunisia Douiret
TP53: Tumor protein p53
TSI: Toscani people of Italy
USF1: Upstream transcription factor 1
USF2: Upstream transcription factor 2, c-fos interacting
VEP: Variant effect predictor
