# Supplementary material for: Decoding the genetic relationship between Alzheimer’s disease and type 2 diabetes: potential risk variants and future direction for North Africa
Source: Front Aging Neurosci. 2023 Jun 5;15:1114810. doi: 10.3389/fnagi.2023.1114810 (PMC10277480; doi:10.3389/fnagi.2023.1114810)
Supplement: Supplementary file 1 [file Table_1.docx]

**Supplementary File 1: Summary of included studies and extracted variants and genes for**

**Decoding the genetic relationship between Alzheimer’s disease and Type 2 Diabetes: Potential risk variants and future direction for North Africa**

Wided Boukhalfa^1,2,3^, Haifa Jmel^1,2^, Nadia Kheriji^1,2,3^, Ismail Gouiza^1,2,3,4^, Hamza Dalleli^1,2^, Mariem Hechmi^1,2^, Rym Kefi^1,2*^

^1^ Laboratory of Biomedical Genomics and Oncogenetics, Institut Pasteur de Tunis, Tunisia

^2^ University of Tunis El Manar Tunis, Tunisia

^3^ Faculty of Medicine of Tunis, Tunis, Tunisia

^4^ University of Angers, MitoLab Team, Unité MitoVasc, UMR CNRS 6015, INSERM U1083, SFR ICAT, Angers, France.

[*rym.kefi@pasteur.utm.tn](mailto:*rym.kefi@pasteur.utm.tn)

**Supplementary Table 1: Summary of 59 studies included in the review**

**Supplementary Table 2: List of 231 extracted variants**

**Supplementary Table 3: List 363 extracted genes**

**TABLE 1: Summary of the 59 studies included in the review**

| **PMID** | **Sample Size** | **Participants Ancestry** | **Dataset/ Experimental Approach** |
| --- | --- | --- | --- |
| 34813915 | 61 | American, European | Expression Profiling By Array |
| 32676761 | Unknown | Unknown | Genetic Database (Omim) |
| 33192474 | 721 | American Indian Or Alaskan Native, Asian, Black Or African American | Expression Profiling By Array |
| 32477452 | Unknown | European, South Asian | Genetic Database |
| 32781959 | Unknown | Unknown | Expression Profiling By Array |
| 32925069 | 2726 | European, Israel, Asian, American | Gwass And Expression Profiling By Array |
| 31849586 | 691 | European, African American, Hispanic, Mixed Ethnicities, | Expression Profiling By Array |
| 29338921 | 355 | American Indian Or Alaskan Native, Asian, Black Or African American | GWAS |
| 32139775 | 448 | Non-Latino White, African American, Latinos | GWAS |
| 29441491 | 3007 | European, North American | GWAS |
| 26947052 | 848 | Israil Jewesh | Genotypage |
| 35996379 | 89 | European And American | Expression Profiling By Array |
| 33226368 | 36 | Turkish | Genotypage |
| 24081379 | 163 | European | Genotypage |
| 30413934 | 79381 | European And North American | GWAS |
| 35570489 | 590 | Chinese | GWAS |
| 28870582 |  | European | Genotypage |
| 26611832 | 2328 | Han Chinese | Genotypage |
| 27516739 | 231 | Han Chinese | Genotypage |
| 35236268 | 1670 | European | Genotypage |
| 24355596 | 97 | Mexico | Genotypage |
| 26178916 | 574 | Chinese Han | High-Throughput Sequencing |
| 23279143 | 220 | Han Chinese | Genotypage |
| 30086706 | 707 | Chinese | Genotypage |
| 27553771 | 8840 | Korean | Genotypage |
| 30861027 | 92 | Mexican American | Genotypage |
| 32818802 | 337484 | European | Phewas |
| 11916953 | 2574 | Japanese-American | Genotypage |
| 30890475 | 301 | Chineese | Genotypage |
| 26639962 | 157488 | European, American, Asian, Middle Eastern, North African | GWAS |
| 26116273 | 333092 Individuals And 3324 Tissues | European | Gwass And E-QTL Datasets |
| 27077117 | 694 | Chinese | Genotypage |
| 33110346 | review | review | review |
| 32739014 | review | review | review |
| 23021038 | review | review | review |
| 30833374 | 1289 | European, American, Asian, Middle Eastern, North African | Protein dosage |
| 34746146 | 852 | Chineese | Protein dosage |
| 33299334 | 205 | Chineese | Protein dosage |
| 26796431 | 50 | European | Protein dosage and APOEε4 genotypage |
| 29255789 | 812 | European, American, Asian, Middle Eastern, North African | Protein dosage |
| 32034441 | 2126 | Chineese | Protein dosage |
| 29951057 | review | review | review |
| 25585531 | 18 | Israel | exome-wide DNA methylation |
| 26745632 | 167 | Han Chinese | Protein dosage and RAGE Gly82Ser genotypage |
| 28777829 | 20 | European | gene expression microarray |
| 32909269 | review | review | review |
| 34528736 | 83 | Chineese | Protein dosage |
| 36506101 | 110 | Chineese | Protein dosage |
| 30747415 | review | review | review |
| 33931404 | 63 | Caucasian and South Asian | Protein dosage |
| 32525932 | 36 | Israel | Methylation450 bead chip |
| 24688222 | 100 | Chineese | Dosage of S-adenosylmethionine/S-adenosylhomocysteine ratio |
| 25793257 | 8 | Chineese |  |
| 26333802 | 816 | European, American, Asian, Middle Eastern, North African | Protein dosage |
| 30429533 | 32 | South African | Protein dosage |
| 28106562 | 388 | Australian | Protein dosage |
| 27079723 | 70 | American | Protein dosage and APOEε4 genotypage |
| 28852028 | 1264 | Australian | Protein dosage and APOEε4 genotypage |
| 20061608 | 198 | Korean | Protein dosage |

**TABLE 2 : List of the 231 extracted variants**

| **Mapped Gene** | **rsID** | **chr** | **Position** | **Allele** | **Variation_type** |
| --- | --- | --- | --- | --- | --- |
| ***AGT*** | rs4762 | chr 1 | 230845977 | G>A | Coding region variation |
| ***AGT*** | rs699 | chr 1 | 230845794 | A>G | Coding region variation |
| ***CELSR2*** | rs629301 | chr 1 | 109818306 | G>T | Non-coding variation |
| ***CELSR2*** | rs646776 | chr 1 | 109818530 | C>T | Non-coding variation |
| ***CFH*** | rs1061170 | chr 1 | 196659237 | C>T | Coding region variation |
| ***CFH*** | rs800292 | chr 1 | 196642233 | G>A | Coding region variation |
| ***CR1*** | rs2296160 | chr 1 | 207795320 | A>G | Coding region variation |
| ***DNM3*** | rs450492 | chr 1 | 172334818 | T>A,C | Non-coding variation |
| ***DNM3*** | rs7539972 | chr 1 | 172342199 | G>A | Non-coding variation |
| ***IL6R*** | rs2228145 | chr 1 | 154426970 | A>C,T | Coding region variation |
| ***LEPR*** | rs1137100 | chr 1 | 66036441 | A>G | Coding region variation |
| ***LEPR*** | rs1137101 | chr 1 | 66058513 | A>G | Coding region variation |
| ***MTHFR*** | rs1801133 | chr 1 | 11856378 | G>A | Coding region variation |
| ***PRDM16*** | rs948081932 | chr 1 | 3122646 | C>T | Non-coding variation |
| ***-*** | rs10169916 | chr 2 | 113603728 | T>A,G | Non-coding variation |
| ***-*** | rs13013349 | chr 2 | 113600326 | T>C | Non-coding variation |
| ***-*** | rs6733839 | chr 2 | 127892810 | C>T | Non-coding variation |
| ***CAPN10*** | rs2975760 | chr 2 | 241531163 | T>C | Non-coding variation |
| ***CAPN10*** | rs3792273 | chr 2 | 241533030 | T>C | Non-coding variation |
| ***CAPN10*** | rs7607759 | chr 2 | 241536126 | A>G | Coding region variation |
| ***GCKR*** | rs1260326 | chr 2 | 27730940 | T>C | Coding region variation |
| ***IL1B*** | rs1143627 | chr 2 | 113594387 | G>A | Non-coding variation |
| ***IL1B*** | rs16944 | chr 2 | 113594867 | A>G | Non-coding variation |
| ***IRS1*** | rs1801278 | chr 2 | 227660544 | C>G,T | Coding region variation |
| ***ADCY5*** | rs2877709 | chr 3 | 123100304 | C>T | Non-coding variation |
| ***AHSG*** | rs4917 | chr 3 | 186337713 | T>C | Coding region variation |
| ***AHSG*** | rs4918 | chr 3 | 186338382 | G>A,C | Coding region variation |
| ***CAMK1*** | rs1052133 | chr 3 | 9798773 | C>G | Coding region variation |
| ***CCR2*** | rs1799864 | chr 3 | 46399208 | G>A | Coding region variation |
| ***CX3CR1*** | rs3732378 | chr 3 | 39307162 | G>A | Coding region variation |
| ***DRD3*** | rs6280 | chr 3 | 113890815 | C>T | Coding region variation |
| ***GHRL*** | rs4684677 | chr 3 | 10328453 | T>A | Coding region variation |
| ***PPARG*** | rs1801282 | chr 3 | 12393125 | C>G | Coding region variation |
| ***PPARG*** | rs1805192 | chr 3 | 12421238 | C>G | Coding region variation |
| ***PPARG*** | rs6809832 | chr 3 | 12414420 | T>C | Non-coding variation |
| ***RHOA*** | rs1050450 | chr 3 | 49394834 | G>A | Coding region variation |
| ***RHOA*** | rs11715915 | chr 3 | 49455330 | C>A,T | Coding region variation |
| ***RHOA*** | rs6997 | chr 3 | 49453834 | C>T | Non-coding variation |
| ***RHOA*** | rs9814873 | chr 3 | 49454112 | A>G | Non-coding variation |
| ***ABCG2*** | rs2231142 | chr 4 | 89052323 | G>C,T | Coding region variation |
| ***CISD2*** | rs223334 | chr 4 | 103788211 | A>G,T | Non-coding variation |
| ***UBE2D3*** | rs150893 | chr 4 | 103738232 | G>A,C,T | Non-coding variation |
| ***UBE2D3*** | rs223387 | chr 4 | 103749516 | C>G,T | Non-coding variation |
| ***UBE2D3*** | rs223452 | chr 4 | 103711434 | C>T | Non-coding variation |
| ***UBE2D3*** | rs223454 | chr 4 | 103710930 | G>A | Non-coding variation |
| ***ADAM19*** | rs7729274 | chr 5 | 156935908 | C>T | Non-coding variation |
| ***AGPAT1*** | rs3132965 | chr 6 | 32146997 | A>G | Non-coding variation |
| ***AGPAT1*** | rs3134940 | chr 6 | 32149816 | T>C | Non-coding variation |
| ***AGPAT1*** | rs3134945 | chr 6 | 32146492 | C>A | Non-coding variation |
| ***AGPAT1*** | rs3134947 | chr 6 | 32145205 | C>T | Non-coding variation |
| ***AGPAT1*** | rs8365 | chr 6 | 32148403 | G>C | Non-coding variation |
| ***HFE*** | rs1799945 | chr 6 | 26091179 | C>G | Coding region variation |
| ***HFE*** | rs1800562 | chr 6 | 26093141 | G>A | Coding region variation |
| ***HLA-DRB9*** | rs7755212 | chr 6 | 32441408 | C>T | Non-coding variation |
| ***HLA-DRB9*** | rs9269078 | chr 6 | 32440879 | C>G,T | Non-coding variation |
| ***LTA*** | rs1799724 | chr 6 | 31542482 | C>T | Non-coding variation |
| ***LTA*** | rs1800629 | chr 6 | 31543031 | G>A | Non-coding variation |
| ***NFKBIL1*** | rs6916921 | chr 6 | 31520426 | C>T | Non-coding variation |
| ***PBX2*** | rs2070600 | chr 6 | 32151443 | C>T | Coding region variation |
| ***PPT2*** | rs3096697 | chr 6 | 32134510 | G>A | Coding region variation |
| ***PPT2*** | rs3130347 | chr 6 | 32134656 | T>C | Non-coding variation |
| ***PRRT1*** | rs3096696 | chr 6 | 32122472 | C>A | Coding region variation |
| ***SUMO2P17*** | rs9269080 | chr 6 | 32440969 | G>A | Non-coding variation |
| ***-*** | rs12534038 | chr 7 | 80329100 | C>G | Non-coding variation |
| ***_*** | rs1262069489 | chr 7 | 66508053 | C>T | Non-coding variation |
| ***IL6*** | rs1474347 | chr 7 | 22768124 | C>A | Non-coding variation |
| ***IL6*** | rs2069832 | chr 7 | 22767433 | A>G,T | Non-coding variation |
| ***MUC12*** | rs1792757405 | chr 7 | 100968871 | C>G,T | Non-coding variation |
| ***NOS3*** | rs1799983 | chr 7 | 150696111 | T>A,G | Coding region variation |
| ***PAX4*** | rs2233580 | chr 7 | 127253550 | C>T | Coding region variation |
| ***PON1*** | rs662 | chr 7 | 94937446 | T>C | Coding region variation |
| ***PON1*** | rs854560 | chr 7 | 94946084 | A>C,G,N,T | Coding region variation |
| ***PON2*** | rs12026 | chr 7 | 95041016 | G>C | Coding region variation |
| ***PON2*** | rs7493 | chr 7 | 95034775 | G>C | Coding region variation |
| ***POR*** | rs1057868 | chr 7 | 75615006 | C>T | Coding region variation |
| ***POR*** | rs17685 | chr 7 | 75616105 | G>A,C | Non-coding variation |
| ***STYXL1*** | rs3823882 | chr 7 | 75631913 | T>A,C | Non-coding variation |
| ***STYXL1*** | rs7785025 | chr 7 | 75628101 | C>A,T | Non-coding variation |
| ***STYXL1*** | rs7807647 | chr 7 | 75628669 | G>A,C | Non-coding variation |
| ***WNT16*** | rs2707466 | chr 7 | 120979089 | C>G,T | Coding region variation |
| ***WNT16*** | rs2908004 | chr 7 | 120969769 | G>A,T | Coding region variation |
| ***-*** | rs1569209 | chr 8 | 19830170 | T>G | Non-coding variation |
| ***-*** | rs17411031 | chr 8 | 19852310 | C>G | Non-coding variation |
| ***-*** | rs17411045 | chr 8 | 19852362 | T>C | Non-coding variation |
| ***-*** | rs17482753 | chr 8 | 19832646 | G>T | Non-coding variation |
| ***-*** | rs17489268 | chr 8 | 19852045 | T>A | Non-coding variation |
| ***-*** | rs17489282 | chr 8 | 19852518 | C>T | Non-coding variation |
| ***-*** | rs1837842 | chr 8 | 19868290 | T>C | Non-coding variation |
| ***-*** | rs1919484 | chr 8 | 19869676 | G>A | Non-coding variation |
| ***ANK1*** | rs515071 | chr 8 | 41519462 | A>G,T | Non-coding variation |
| ***CLU*** | rs11136000 | chr 8 | 27464519 | T>C | Non-coding variation |
| ***EPHX2*** | rs751141 | chr 8 | 27373865 | G>A | Coding region variation |
| ***HLA-DRB9*** | rs896854 | chr 8 | 94948283 | T>A,C,G | Non-coding variation |
| ***LPL*** | rs12679834 | chr 8 | 19820433 | T>C | Non-coding variation |
| ***LPL*** | rs326 | chr 8 | 19819439 | A>G | Non-coding variation |
| ***LPL*** | rs327 | chr 8 | 19819536 | T>G | Non-coding variation |
| ***NDUFAF6*** | rs4734295 | chr 8 | 94988691 | A>G,T | Non-coding variation |
| ***NDUFAF6*** | rs6982393 | chr 8 | 95980708 | T>A,C | Non-coding variation |
| ***NDUFAF6*** | rs7812465 | chr 8 | 96050557 | T>C | Non-coding variation |
| ***TP53INP1*** | rs896854 | chr 8 | 95960511 | T>C | Non-coding variation |
| ***ABCA1*** | rs1800977 | chr 9 | 107690450 | G>A,C | Non-coding variation |
| ***ABCA1*** | rs2066714 | chr 9 | 107586753 | T>C | Coding region variation |
| ***ABCA1*** | rs2230806 | chr 9 | 107620867 | C>T | Coding region variation |
| ***ABCA1*** | rs2230808 | chr 9 | 107562804 | T>C | Coding region variation |
| ***-*** | rs10510109 | chr 10 | 124120457 | G>A,T | Non-coding variation |
| ***-*** | rs1544210 | chr 10 | 94487801 | G>A | Non-coding variation |
| ***BORCS7*** | rs4409766 | chr 10 | 104616663 | T>C | Non-coding variation |
| ***BORCS7*** | rs7096475 | chr 10 | 104609676 | T>C | Non-coding variation |
| ***IDE*** | rs2421943 | chr 10 | 94311815 | G>A | Non-coding variation |
| ***IDE*** | rs6583817 | chr 10 | 94247247 | C>T | Non-coding variation |
| ***LIPA*** | rs1051338 | chr 10 | 91007360 | T>G | Coding region variation |
| ***LIPA*** | rs1412444 | chr 10 | 91002927 | C>T | Non-coding variation |
| ***LIPA*** | rs2246833 | chr 10 | 91005854 | C>T | Non-coding variation |
| ***MARK2P9*** | rs2209972 | chr 10 | 94179028 | C>T | Non-coding variation |
| ***PLEKHA1*** | rs2421016 | chr 10 | 124167512 | C>T | Non-coding variation |
| ***SORCS1*** | rs7907690 | chr 10 | 108862127 | A>G | Non-coding variation |
| ***WBP1L*** | rs3781287 | chr 10 | 104595420 | T>G | Non-coding variation |
| ***WBP1L*** | rs4919683 | chr 10 | 104585125 | C>A,T | Non-coding variation |
| ***ACP2*** | rs2167079 | chr 11 | 47270255 | C>T | Coding region variation |
| ***ANKK1*** | rs1800497 | chr 11 | 113270828 | G>A | Coding region variation |
| ***ANKK1*** | rs7118900 | chr 11 | 113266821 | G>A | Coding region variation |
| ***AP2A2*** | rs7396366 | chr 11 | 986185 | C>A | Non-coding variation |
| ***BDNF*** | rs6265 | chr 11 | 27679916 | C>T | Coding region variation |
| ***DDB2*** | rs326222 | chr 11 | 47259668 | T>C | Non-coding variation |
| ***DDB2*** | rs830085 | chr 11 | 47251199 | G>A,C | Non-coding variation |
| ***GSTP1*** | rs1695 | chr 11 | 67352689 | A>G | Coding region variation |
| ***MADD*** | rs10501321 | chr 11 | 47294626 | T>C | Non-coding variation |
| ***MADD*** | rs1052373 | chr 11 | 47354787 | C>T | Coding region variation |
| ***MADD*** | rs10838692 | chr 11 | 47345100 | T>C | Non-coding variation |
| ***MADD*** | rs1375688 | chr 11 | 47327217 | G>A | Non-coding variation |
| ***MADD*** | rs1449627 | chr 11 | 47290984 | T>C,G | Non-coding variation |
| ***MADD*** | rs3781622 | chr 11 | 47348702 | T>C | Non-coding variation |
| ***MADD*** | rs4752824 | chr 11 | 47324666 | T>A | Non-coding variation |
| ***MADD*** | rs7120118 | chr 11 | 47286290 | T>C | Non-coding variation |
| ***MADD*** | rs7124955 | chr 11 | 47315274 | T>A | Non-coding variation |
| ***MMP3*** | rs679620 | chr 11 | 102713620 | T>C | Coding region variation |
| ***ZPR1*** | rs651821 | chr 11 | 116662579 | C>T | Non-coding variation |
| ***MPHOSPH9*** | rs1716168 | chr 12 | 123651966 | G>C | Non-coding variation |
| ***MPHOSPH9*** | rs1727315 | chr 12 | 123638930 | G>A,T | Non-coding variation |
| ***MUC19*** | rs7302859 | chr 12 | 40957918 | G>A | Non-coding variation |
| ***PITPNM2*** | rs1106240 | chr 12 | 123626982 | C>T | Non-coding variation |
| ***PITPNM2*** | rs1569068 | chr 12 | 123632276 | G>A | Non-coding variation |
| ***PITPNM2*** | rs1790116 | chr 12 | 123618544 | G>T | Non-coding variation |
| ***AKT1*** | rs2498786 | chr 14 | 105262368 | C>G | Non-coding variation |
| ***DIO2*** | rs225014 | chr 14 | 80669580 | T>C | Coding region variation |
| ***ESR2*** | rs1256033 | chr 14 | 64745399 | T>A,C | Non-coding variation |
| ***OSGEP*** | rs1130409 | chr 14 | 20925154 | T>A,C,G | Coding region variation |
| ***-*** | rs2036527 | chr 15 | 78851615 | G>A | Non-coding variation |
| ***PML*** | rs12902857 | chr 15 | 74317871 | C>G | Non-coding variation |
| ***PML*** | rs2304717 | chr 15 | 74326557 | T>C | Non-coding variation |
| ***PML*** | rs3784562 | chr 15 | 74291023 | G>A,C | Non-coding variation |
| ***CBFA2T3*** | rs1230970038 | chr 16 | 88967884 | C>T | Non-coding variation |
| ***CETP*** | rs1532625 | chr 16 | 57005301 | C>T | Non-coding variation |
| ***UMOD*** | rs13335818 | chr 16 | 20359831 | C>A,T | Coding region variation |
| ***UMOD*** | rs4293393 | chr 16 | 20364588 | A>G | Non-coding variation |
| ***-*** | rs9904761 | chr 17 | 46956830 | C>G | Non-coding variation |
| ***_*** | rs889449553 | chr 17 | 69907648 | C>T | Non-coding variation |
| ***ATP5MC1*** | rs1962412 | chr 17 | 46970259 | T>C,G | Non-coding variation |
| ***CALCOCO2*** | rs10278 | chr 17 | 46939658 | C>A,G | Coding region variation |
| ***EFCAB5*** | rs11080114 | chr 17 | 28407287 | C>T | Non-coding variation |
| ***EFCAB5*** | rs4350617 | chr 17 | 28423088 | G>A | Non-coding variation |
| ***EFCAB5*** | rs4598962 | chr 17 | 28293948 | C>A,G,T | Non-coding variation |
| ***EFCAB5*** | rs7212497 | chr 17 | 28335949 | T>A,G | Non-coding variation |
| ***EFCAB5*** | rs7221743 | chr 17 | 28407876 | T>G | Coding region variation |
| ***EFCAB5*** | rs7222308 | chr 17 | 28277041 | T>C | Non-coding variation |
| ***EFCAB5*** | rs8065059 | chr 17 | 28417376 | T>C | Non-coding variation |
| ***EFCAB5*** | rs9897794 | chr 17 | 28296327 | T>C,G | Coding region variation |
| ***EFCAB5*** | rs9902453 | chr 17 | 28349095 | G>A | Non-coding variation |
| ***NSRP1*** | rs1906451 | chr 17 | 28515479 | A>G | Non-coding variation |
| ***NSRP1*** | rs4429345 | chr 17 | 28458105 | A>G | Non-coding variation |
| ***RNY4P13*** | rs9890205 | chr 17 | 28382704 | C>T | Non-coding variation |
| ***SREBF1*** | rs4925115 | chr 17 | 17721457 | A>G | Non-coding variation |
| ***SREBF1*** | rs9899634 | chr 17 | 17727943 | T>A,C | Non-coding variation |
| ***SRR*** | rs391300 | chr 17 | 2216258 | T>C | Non-coding variation |
| ***SUMO2P17*** | rs962273 | chr 17 | 46978353 | T>C | Non-coding variation |
| ***SUMO2P17*** | rs4793990 | chr 17 | 46960760 | G>A | Non-coding variation |
| ***TBC1D16*** | rs770003816 | chr 17 | 80010547 | C>T | Coding region variation |
| ***TP53*** | rs11540654 | chr 17 | 7579358 | C>A,G,T | Coding region variation |
| ***KCNG2*** | rs552447248 | chr 18 | 79878446 | C>T | Non-coding variation |
| ***LIPG*** | rs2000813 | chr 18 | 47093864 | C>T | Coding region variation |
| ***ABCA7*** | rs3752246 | chr 19 | 1056493 | G>C,T | Non-coding variation |
| ***ABCA7*** | rs4147929 | chr 19 | 1063444 | A>C,G,T | Non-coding variation |
| ***APOC1*** | rs12721046 | chr 19 | 45421254 | G>A | Non-coding variation |
| ***APOC1*** | rs12721051 | chr 19 | 45422160 | C>G | Non-coding variation |
| ***APOC1*** | rs4420638 | chr 19 | 45422946 | A>G | Non-coding variation |
| ***APOC1*** | rs56131196 | chr 19 | 45422846 | G>A | Non-coding variation |
| ***APOC1P1*** | rs111789331 | chr 19 | 45427125 | T>A | Non-coding variation |
| ***APOC1P1*** | rs66626994 | chr 19 | 45428234 | G>A | Non-coding variation |
| ***APOC2*** | rs2288911 | chr 19 | 45449284 | T>G | Non-coding variation |
| ***APOC2*** | rs7257476 | chr 19 | 45453165 | C>T | Non-coding variation |
| ***APOE*** | rs405509 | chr 19 | 45408836 | T>G | Non-coding variation |
| ***APOE*** | rs429358 | chr 19 | 45411941 | T>C | Coding region variation |
| ***APOE*** | rs439401 | chr 19 | 45414451 | T>C | Non-coding variation |
| ***APOE*** | rs584007 | chr 19 | 45416478 | A>G | Non-coding variation |
| ***APOE*** | rs7412 | chr 19 | 45412079 | C>T | Coding region variation |
| ***B9D2*** | rs2241713 | chr 19 | 41869468 | G>A,C | Non-coding variation |
| ***B9D2*** | rs2241714 | chr 19 | 41869392 | T>A,C | Coding region variation |
| ***CLPTM1*** | rs11669173 | chr 19 | 45492559 | G>A | Non-coding variation |
| ***CLPTM1*** | rs2239375 | chr 19 | 45459851 | T>C | Non-coding variation |
| ***CLPTM1*** | rs3760629 | chr 19 | 45458146 | A>C | Non-coding variation |
| ***ICAM1*** | rs1799969 | chr 19 | 10394792 | G>A | Coding region variation |
| ***ICAM5*** | rs5498 | chr 19 | 10395683 | A>G | Coding region variation |
| ***NECTIN2*** | rs10426423 | chr 19 | 45348253 | C>A,G,T | Non-coding variation |
| ***NECTIN2*** | rs157580 | chr 19 | 45395266 | G>A | Non-coding variation |
| ***NECTIN2*** | rs1871047 | chr 19 | 45351746 | A>G | Non-coding variation |
| ***NECTIN2*** | rs2075650 | chr 19 | 45395619 | A>G | Non-coding variation |
| ***NECTIN2*** | rs2306149 | chr 19 | 45349963 | C>A | Non-coding variation |
| ***NECTIN2*** | rs6859 | chr 19 | 45382034 | A>G | Non-coding variation |
| ***TGFB1*** | rs1800469 | chr 19 | 41860296 | A>G | Non-coding variation |
| ***TGFB1*** | rs1800470 | chr 19 | 41858921 | G>A,C | Coding region variation |
| ***TGFB1*** | rs1982072 | chr 19 | 41864509 | T>A | Non-coding variation |
| ***TOMM40*** | rs1160985 | chr 19 | 45403412 | C>T | Non-coding variation |
| ***-*** | rs4815222 | chr 20 | 23576964 | G>A | Non-coding variation |
| ***-*** | rs4815224 | chr 20 | 23594112 | G>C | Non-coding variation |
| ***-*** | rs6036461 | chr 20 | 23576754 | G>C | Non-coding variation |
| ***-*** | rs6048920 | chr 20 | 23576844 | T>C | Non-coding variation |
| ***CST3*** | rs1011226 | chr 20 | 23605833 | A>C | Non-coding variation |
| ***CST3*** | rs1064039 | chr 20 | 23618427 | C>G,T | Coding region variation |
| ***CST3*** | rs12625716 | chr 20 | 23606845 | G>A | Non-coding variation |
| ***CST3*** | rs2254635 | chr 20 | 23622758 | T>C,G | Non-coding variation |
| ***CST3*** | rs2405367 | chr 20 | 23622880 | G>A | Non-coding variation |
| ***CST3*** | rs2424582 | chr 20 | 23622550 | G>A | Non-coding variation |
| ***CST9*** | rs3004145 | chr 20 | 23582876 | C>G | Non-coding variation |
| ***CST9*** | rs6036465 | chr 20 | 23588003 | G>T | Non-coding variation |
| ***PLCG1*** | rs753381 | chr 20 | 39797465 | T>C | Coding region variation |
| ***COMT*** | rs4680 | chr 22 | 19951271 | G>A | Coding region variation |
| ***PPARA*** | rs1800206 | chr 22 | 46614274 | C>G | Coding region variation |
| ***PLPPR4*** | rs1658823313 | chr 1 | 99263904 | C>A | Non-coding variation |
| ***MUC12*** | rs1792757405 | chr 7 | 100968871 | C>G,T | Non-coding variation |
| ***GLYATL2*** | rs1189882276 | chr 11 | 58905980 |  | Non-coding variation |
| ***CD33*** | rs3865444 | chr 19 | 51224706 |  | Non-coding variation |

*- : variants in intergenic region

**TABLE 3: List of the 363 extracted genes**

| Gene Symbole | **Chromosome** | **Start Position in Kb** | **End Position in Kb** |
| --- | --- | --- | --- |
| *ADSS* | 1 | 104729 | 104747 |
| ***AGT*** | 1 | 230702 | 230745 |
| *C1QB* | 1 | 22652 | 22661 |
| *CELSR2* | 1 | 109249 | 109275 |
| *CFH* | 1 | 196652 | 196747 |
| *CR1* | 1 | 207496 | 207641 |
| *DNM3* | 1 | 171817 | 172418 |
| *HSD11B1* | 1 | 209686 | 209734 |
| *IFI16* | 1 | 158999 | 159055 |
| ***IL6R*** | 1 | 154405 | 154469 |
| *LEPR* | 1 | 65420 | 64641 |
| *MTHFR* | 1 | 11785 | 11803 |
| *PPAP2B* | 1 | 56494 | 56645 |
| *PPEF1* | 1 | 31629 | 31644 |
| *PTPN22* | 1 | 113813 | 13871 |
| *S100A8* | 1 | 153390 | 153391 |
| *SFN* | 1 | 26863 | 26864 |
| *TIPRL* | 1 | 168178 | 168202 |
| *TSNAX* | 1 | 231582 | 231566 |
| *CACNA1S* | 1 | 201039 | 201112 |
| *CRP* | 1 | 159712 | 159714 |
| *LPPR4* | 1 | 99264 | 99309 |
| *PRDM16* | 1 | 3069 | 3438 |
| *PRKAA2* | 1 | 56645 | 56715 |
| *PSEN2* | 1 | 226870 | 226927 |
| *RUSC1* | 1 | 155320 | 155331 |
| *RXFP4* | 1 | 155941 | 155943 |
| *VCAM1* | 1 | 100719 | 100739 |
| *WNT3A* | 1 | 228006 | 228061 |
| *DPYD* | 1 | 97077 | 97995 |
| ***F5*** | 1 | 169511 | 169586 |
| ***DAB1*** | 1 | 56994 | 58546 |
| ***BIN1*** | 2 | 127048 | 127107 |
| *C2orf80* | 2 | 208165 | 208190 |
| *CAPN10* | 2 | 240586 | 240599 |
| ***GCKR*** | 2 | 26496 | 27523 |
| *GLS* | 2 | 190880 | 190965 |
| *IL1B* | 2 | 112829 | 112836 |
| *IRS1* | 2 | 226731 | 226799 |
| *ITGA4* | 2 | 181457 | 181538 |
| *LINC01123* | 2 | 109987 | 109996 |
| *MERTK* | 2 | 111898 | 112029 |
| *OLA1* | 2 | 174072 | 174248 |
| *PPM1B* | 2 | 44167 | 44244 |
| *TIA1* | 2 | 70209 | 70248 |
| *ZBTB45P1* | 2 | 109986 | 109988 |
| *APOB* | 2 | 21001 | 21044 |
| *GREB1* | 2 | 11482 | 11642 |
| *XRCC5* | 2 | 216107 | 216206 |
| *SNRNP27* | 2 | 69893 | 96905 |
| *SOS1* | 2 | 38962 | 39124 |
| *CNTNAP5* | 2 | 124025 | 124921 |
| *CCDC148* | 2 | 158171 | 158456 |
| *ADCY5* | 3 | 123282 | 123449 |
| *AHSG* | 3 | 186613 | 186621 |
| *AMT* | 3 | 49416 | 49422 |
| *CAMK1* | 3 | 9757 | 9769 |
| *CCR2* | 3 | 46354 | 46360 |
| *CX3CR1* | 3 | 39263 | 39281 |
| *DRD3* | 3 | 186613 | 186621 |
| *GATA2* | 3 | 128479 | 128493 |
| *GHRL* | 3 | 10285 | 10292 |
| *GHRLOS* | 3 | 10280 | 10293 |
| *GPX1* | 3 | 49357 | 49358 |
| *HIGD1A* | 3 | 42782 | 42804 |
| *MRAS* | 3 | 138347 | 138405 |
| *NMD3* | 3 | 161104 | 161253 |
| *OGG1* | 3 | 9749 | 9766 |
| *PPARG* | 3 | 12351 | 12410 |
| *RYK* | 3 | 134157 | 134250 |
| *SLC25A36* | 3 | 140941 | 140980 |
| *SLC6A1* | 3 | 10992 | 11039 |
| *SST* | 3 | 187668 | 187670 |
| *ZNF654* | 3 | 88059 | 88144 |
| *ADIPOQ* | 3 | 186842 | 186858 |
| *CIP2A* | 3 | 108549 | 108589 |
| *CP* | 3 | 149162 | 149221 |
| ***GSK3β*** | 3 | 119821 | 120094 |
| *ITIH1* | 3 | 52777 | 52792 |
| *RHOA* | 3 | 49359 | 49412 |
| *ACAD11* | 3 | 132558 | 132660 |
| *TP63* | 3 | 189631 | 189897 |
| *ABCG2* | 4 | 88090 | 88231 |
| *COMMD8* | 4 | 47450 | 47463 |
| *DCK* | 4 | 70992 | 71030 |
| *DNAJB14* | 4 | 99896 | 99946 |
| *EPHA5* | 4 | 65319 | 65670 |
| *G3BP2* | 4 | 75642 | 75724 |
| *LOC102723704* | 4 | 102777 | 102799 |
| ***OTUD4*** | 4 | 145110 | 145180 |
| *SCD5* | 4 | 82629 | 82798 |
| *TBC1D9* | 4 | 140620 | 140756 |
| *UBE2D3* | 4 | 102794 | 102686 |
| *CAMK2D* | 4 | 113418 | 113761 |
| *CISD2* | 4 | 102868 | 102892 |
| ***INPP4B*** | 4 | 142023 | 142847 |
| *ADAM19* | 5 | 157395 | 157575 |
| *ANKH* | 5 | 14704 | 14871 |
| *CARTPT* | 5 | 71719 | 71721 |
| *COMMD10* | 5 | 116085 | 116190 |
| *GTF2H2C* | 5 | 69560 | 69595 |
| *MRPS30* | 5 | 44808 | 44820 |
| *NAIP* | 5 | 70968 | 71025 |
| *PCSK1* | 5 | 96390 | 96434 |
| *SERF1B* | 5 | 70025 | 70043 |
| *SMN2* | 5 | 70049 | 70078 |
| *TCEA1* | 5 | 53966 | 54022 |
| *TMED7* | 5 | 115613 | 115626 |
| *TNPO1* | 5 | 72816 | 72916 |
| *ADRA1B* | 5 | 159865 | 159973 |
| *CRHBP* | 5 | 76953 | 76981 |
| *KCNIP1* | 5 | 170353 | 170736 |
| *KCNMB1* | 5 | 170374 | 170389 |
| *PPP2CA* | 5 | 134194 | 134226 |
| *TIMD4* | 5 | 156919 | 156963 |
| *GHR* | 5 | 42423 | 42721 |
| ***ADAMTS12*** | 5 | 33523 | 33892 |
| ***SLIT3*** | 5 | 168661 | 169301 |
| ***AGER*** | 6 | 32180 | 32184 |
| *BCLAF1* | 6 | 136256 | 136289 |
| *BTNL2* | 6 | 32393 | 32407 |
| *C6ORF10* | 6 | 32292 | 32371 |
| *CCNC* | 6 | 99542 | 99568 |
| *CD2AP* | 6 | 47477 | 47627 |
| *EGFL8* | 6 | 32164 | 32168 |
| *FOXC1* | 6 | 1609 | 1613 |
| *GJA1* | 6 | 121435 | 121449 |
| ***HFE*** | 6 | 26087 | 26098 |
| *HIST1H2BJ* | 6 | 27125 | 27132 |
| *LAMA4* | 6 | 112107 | 112254 |
| *LOC100507547* | 6 | 32152 | 32154 |
| *LPA* | 6 | 160531 | 160664 |
| *MAP3K7* | 6 | 90513 | 90587 |
| *MED23* | 6 | 131573 | 131628 |
| *PPT2* | 6 | 32154 | 32162 |
| *PPT2-EGFL8* | 6 | 32153 | 32168 |
| *PRPF4B* | 6 | 4021 | 4064 |
| *PRRT1* | 6 | 32148 | 32151 |
| *RNF5* | 6 | 32178 | 32180 |
| *SOD2* | 6 | 159669 | 159762 |
| ***TNFA*** | 6 | 31575 | 31578 |
| *VEGFA* | 6 | 43770 | 43786 |
| *AGPAT1* | 6 | 32168 | 326178 |
| *AKD1* | 6 | 109492 | 109691 |
| *AMD1* | 6 | 110874 | 110898 |
| *C4* | 6 | 31982 | 32002 |
| *HLA-DRB9* | 6 | 32459 | 32473 |
| *LTA* | 6 | 31572 | 31574 |
| *MICB* | 6 | 31494 | 31511 |
| *MIR1275* | 6 | 33999 | 34000 |
| *NFKBIL1* | 6 | 31546 | 31558 |
| *PBX2* | 6 | 32184 | 32190 |
| *TRIM31* | 6 | 30102 | 30113 |
| *C6orf138* | 6 | 47856 | 48111 |
| *LOC401397* | 7 | 113116 | 113118 |
| *LRRN3* | 7 | 111091 | 111125 |
| *NOS3* | 7 | 150991 | 151014 |
| *PAX4* | 7 | 127610 | 127618 |
| *PON1* | 7 | 95397 | 95324 |
| *PON2* | 7 | 95404 | 95435 |
| *POR* | 7 | 75915 | 75986 |
| *SP4* | 7 | 21428 | 21514 |
| *STYXL1* | 7 | 75996 | 76048 |
| *WNT16* | 7 | 121329 | 121341 |
| *FSCN3* | 7 | 127591 | 127602 |
| *IL6* | 7 | 22725 | 22732 |
| *KCTD7* | 7 | 66628 | 66649 |
| *MUC12* | 7 | 100969 | 101018 |
| *AGPAT5* | 8 | 6708 | 6761 |
| *ANK1* | 8 | 41653 | 41896 |
| ***CLU*** | 8 | 27596 | 27614 |
| *CNOT7* | 8 | 17224 | 17246 |
| *EPHX2* | 8 | 27491 | 27454 |
| *GEM* | 8 | 94249 | 94262 |
| ***LPL*** | 8 | 19901 | 19967 |
| ***NDUFAF6*** | 8 | 94895 | 95116 |
| *PKIA* | 8 | 78516 | 78605 |
| *RANP9* | 8 | 33039 | 33039 |
| ***TP53INP1*** | 8 | 94925 | 94949 |
| *CTD-3025N20.2* | 8 | 66439 | 66475 |
| *UBE2W* | 8 | 73780 | 73878 |
| *SGCZ* | 8 | 14084 | 15238 |
| *RP1* | 8 | 54509 | 54871 |
| ***ABCA1*** | 9 | 104781 | 104928 |
| *PTPRD* | 9 | 8314 | 10613 |
| *RN7SL763P* | 9 | 39886 | 39887 |
| *TXN* | 9 | 110243 | 110256 |
| *ZNF658B* | 9 | 39443 | 39508 |
| *GARNL3* | 9 | 127224 | 127393 |
| *PAPPA* | 9 | 116153 | 116402 |
| *PTPA2* | 9 | 129110 | 129148 |
| *VCP* | 9 | 35053 | 35072 |
| *ATAD1* | 10 | 87751 | 87841 |
| *BORCS7* | 10 | 10285 | 10286 |
| ***BTBD16*** | 10 | 122271 | 122338 |
| *CYP17A1* | 10 | 102830 | 102837 |
| *HERC4* | 10 | 67921 | 68075 |
| *HHEX* | 10 | 92689 | 92695 |
| ***IDE*** | 10 | 92451 | 92574 |
| *LIPA* | 10 | 98213 | 89251 |
| ***PLEKHA1*** | 10 | 122374 | 122432 |
| *SORCS1* | 10 | 106753 | 107164 |
| *SRP9P1* | 10 | 91807 | 91807 |
| *TCF7L2* | 10 | 112950 | 113167 |
| *ITIH2* | 10 | 7703 | 7749 |
| *MARK2P9* | 10 | 92418 | 92420 |
| *WBP1L* | 10 | 102743 | 102834 |
| ***TACC2*** | 10 | 121989 | 122254 |
| ***SVIL*** | 10 | 29457 | 29736 |
| *FAM35A* | 10 | 87094 | 87191 |
| *ACP2* | 11 | 47239 | 47248 |
| *ANKK1* | 11 | 113387 | 113400 |
| *AP2A2* | 11 | 924 | 1012 |
| *APOA5* | 11 | 116789 | 116792 |
| *ATM* | 11 | 108223 | 108369 |
| ***BDNF*** | 11 | 27654 | 27722 |
| *CAPRIN1* | 11 | 34051 | 34102 |
| *CD44* | 11 | 35138 | 35232 |
| *DDB2* | 11 | 47214 | 47239 |
| *GSTP1* | 11 | 67583 | 67586 |
| *HINFP* | 11 | 119121 | 119136 |
| *INS* | 11 | 2159 | 2161 |
| *MADD* | 11 | 47270 | 47330 |
| *MMP3* | 11 | 102835 | 102843 |
| *MYBPC3* | 11 | 47331 | 47352 |
| *NR1H3* | 11 | 47248 | 47269 |
| *NUP160* | 11 | 47778 | 47848 |
| *PDE3B* | 11 | 14643 | 14872 |
| *SLC22A9* | 11 | 63369 | 63410 |
| *APOA1* | 11 | 116835 | 116837 |
| *APOA4* | 11 | 116820 | 116823 |
| *ARHGAP20* | 11 | 110577 | 110713 |
| *GLYATL2* | 11 | 58834 | 58904 |
| *NAALADL1* | 11 | 65044 | 65058 |
| *PICALM* | 11 | 85957 | 86069 |
| *SAA1* | 11 | 18266 | 18269 |
| *SORL1* | 11 | 121452 | 121633 |
| *ZPR1* | 11 | 116773 | 116788 |
| ***NAV2*** | 11 | 19350 | 20121 |
| ***ZBTB16*** | 11 | 114059 | 114256 |
| ***SCUBE2*** | 11 | 9019 | 9138 |
| *BBS10* | 12 | 76344 | 76348 |
| ***CDKN1B*** | 12 | 12685 | 12722 |
| *CLEC2D* | 12 | 9669 | 9699 |
| *ETNK1* | 12 | 22625 | 22690 |
| ***LRRK2*** | 12 | 40196 | 40369 |
| *MPHOSPH9* | 12 | 123152 | 123244 |
| *MUC19* | 12 | 40393 | 40570 |
| *PITPNM2* | 12 | 122983 | 123151 |
| *SNRPF* | 12 | 95858 | 95903 |
| *VWF* | 12 | 5948 | 6124 |
| *A2M* | 12 | 9067 | 9116 |
| *RAPGEF3* | 12 | 47734 | 47771 |
| *WDR66* | 12 | 121918 | 121003 |
| *SLCO1B1* | 12 | 21131 | 21239 |
| *AKAP11* | 13 | 42272 | 42323 |
| *TRIM13* | 13 | 49997 | 50018 |
| *DIAPH3* | 13 | 59665 | 60163 |
| *FNDC3A* | 13 | 48975 | 49209 |
| *AKT1* | 14 | 104769 | 104795 |
| *AP5M1* | 14 | 57268 | 57298 |
| *APEX1* | 14 | 20455 | 20457 |
| ***CALM1*** | 14 | 90396 | 90408 |
| *DIO2* | 14 | 80197 | 80212 |
| *ESR2* | 14 | 46228 | 46294 |
| *PPM1A* | 14 | 60249 | 60299 |
| ***SERPINA3*** | 14 | 94612 | 94624 |
| *SOCS4* | 14 | 55027 | 55049 |
| *YY1* | 14 | 100238 | 100282 |
| *OSGEP* | 14 | 20446 | 20455 |
| *PPP2R3C* | 14 | 35085 | 35122 |
| *PSEN1* | 14 | 73136 | 73223 |
| *SERPINA1* | 14 | 94376 | 94390 |
| ***SAMD4A*** | 14 | 54566 | 54793 |
| *TSHR* | 14 | 80954 | 81146 |
| *DPF3* | 14 | 72609 | 72894 |
| *BTBD1* | 15 | 83016 | 83067 |
| *CHRNA5* | 15 | 78565 | 78595 |
| *EIF3J* | 15 | 44537 | 44563 |
| *PML* | 15 | 73994 | 74047 |
| *CLN6* | 15 | 68206 | 68257 |
| *IDH3A* | 15 | 78131 | 78717 |
| *RBPMS2* | 15 | 64739 | 64775 |
| *CETP* | 16 | 56961 | 56983 |
| *FOXL1* | 16 | 86576 | 86583 |
| *NUDT21* | 16 | 56429 | 56451 |
| *SNTB2* | 16 | 69187 | 69309 |
| *UMOD* | 16 | 20333 | 20356 |
| *ADCY9* | 16 | 3953 | 4116 |
| *CACNG3* | 16 | 24256 | 24362 |
| *CBFA2T3* | 16 | 88874 | 88977 |
| *HP* | 16 | 72054 | 72061 |
| *RAB40C* | 16 | 589357 | 629272 |
| *AC009019.10* | 16 | 22007 | 22087 |
| ***CDH13*** | 16 | 82626 | 83800 |
| *AC132872.2* | 17 | 82316 | 82317 |
| *ATP5MC1* | 17 | 48892 | 48895 |
| *CALCOCO2* | 17 | 48831 | 48865 |
| *EFCAB5* | 17 | 29941 | 30108 |
| *GFAP* | 17 | 44903 | 44916 |
| *GNA13* | 17 | 65009 | 65056 |
| *LOC105371814* | 17 | 48874 | 48908 |
| *NSRP1* | 17 | 30115 | 30186 |
| *SREBF1* | 17 | 17811 | 17836 |
| ***SRR*** | 17 | 2303 | 2325 |
| *TP53* | 17 | 9757 | 9769 |
| *CCDC144C* | 17 | 20321 | 20403 |
| *EFCAB3* | 17 | 62343 | 62416 |
| *KCNJ16* | 17 | 70053 | 70135 |
| ***MAPT*** | 17 | 45894 | 46028 |
| *PPY* | 17 | 43940 | 43942 |
| *RNY4P13* | 17 | 30059 | 30059 |
| *SUMO2P17* | 17 | 48874 | 48908 |
| *TBC1D16* | 17 | 79932 | 80035 |
| *ABCA8* | 17 | 68867 | 68955 |
| *LIPG* | 18 | 49562 | 49599 |
| *MIB1* | 18 | 21704 | 21870 |
| *RPL17* | 18 | 49488 | 49492 |
| *KCNG2* | 18 | 79797 | 79900 |
| *TTR* | 18 | 31557 | 31598 |
| *RTTN* | 18 | 70003 | 70205 |
| ***APOC1*** | 19 | 44914 | 44919 |
| *APOC2* | 19 | 44946 | 44949 |
| ***APOE*** | 19 | 44905 | 44909 |
| *B9D2* | 19 | 41354 | 41364 |
| *CLPTM1* | 19 | 44954 | 44993 |
| *ICAM1* | 19 | 10271 | 10286 |
| ***ICAM4*** | 19 | 10286 | 10288 |
| ***NECTIN2*** | 19 | 44846 | 44889 |
| *PVRL2* | 19 | 44882 | 44890 |
| *TGFB1* | 19 | 41330 | 41353 |
| *TMEM91* | 19 | 41376 | 41384 |
| ***TOMM40*** | 19 | 44890 | 44903 |
| *ABCA7* | 19 | 1039 | 1065 |
| *APOC1P1* | 19 | 44926 | 44931 |
| ***ICAM5*** | 19 | 10289 | 10296 |
| *TNNI3* | 19 | 55151 | 55157 |
| *ZC3H4* | 19 | 47064 | 47113 |
| ***ZNF480*** | 19 | 52297 | 52325 |
| *CST3* | 20 | 23626 | 263637 |
| *CST9* | 20 | 23602 | 23605 |
| *DPM1* | 20 | 50934 | 50959 |
| *MMP9* | 20 | 46008 | 46016 |
| ***PLCG1*** | 20 | 41137 | 41177 |
| *AHCY* | 20 | 34280 | 34311 |
| *CBLN4* | 20 | 55997 | 56005 |
| *LBP* | 20 | 38346 | 38377 |
| *PRNP* | 20 | 4868 | 4701 |
| *PLCB1* | 20 | 8077 | 8968 |
| *NCRNA00153* | 20 | 21125 | 21246 |
| *ABCG1* | 21 | 42199 | 42297 |
| *GABPA* | 21 | 25734 | 25772 |
| *ITGB2* | 21 | 44885 | 44931 |
| *MORC3* | 21 | 36320 | 36386 |
| *MRPS6* | 21 | 34073 | 34143 |
| ***APP*** | 21 | 25880 | 26171 |
| *COMT* | 22 | 19941 | 19969 |
| *HMOX1* | 22 | 35384 | 35390 |
| *MIR4761* | 22 | 19963 | 19963 |
| *PPARA* | 22 | 46150 | 46243 |
| *RBX1* | 22 | 40951 | 40973 |
| ***KREMEN1*** | 22 | 22073 | 29168 |
| *DUSP9* | X | 153642 | 153651 |

*Replicated genes among studies are marked in bold characters
