# Supplementary material for: Decoding the genetic relationship between Alzheimer’s disease and type 2 diabetes: potential risk variants and future direction for North Africa
Source: Front Aging Neurosci. 2023 Jun 5;15:1114810. doi: 10.3389/fnagi.2023.1114810 (PMC10277480; doi:10.3389/fnagi.2023.1114810)
Supplement: Supplementary file 8 [file Data_Sheet_1.PDF]

Supplementary File 4 Summary of statistical analysis for

Decoding the Genetic Relationship Between Alzheimer's disease and Type 2 Diabetes: Potential Risk Variants and Future Direction for North Africa

Wided Boukhalfa[1,2,3], Haifa Jmel[1,2], Nadia Kheriji[1,2,3], Ismail Gouiza[1,2,3,4], Hamza Dalleli[1,2], Mariem Hechmi[1,2], Rym Kefi[1,2*]

[1] Laboratory of Biomedical Genomics and Oncogenetics, Institut Pasteur de Tunis, Tunisia

[2] University of Tunis El Manar Tunis, Tunisia

[3] Faculty of Medicine of Tunis, Tunis, Tunisia

[4] University of Angers, MitoLab Team, Unité MitoVasc, UMR CNRS 6015, INSERM U1083, SFR ICAT, Angers, France.

*rym.kefi@pasteur.utm.tn

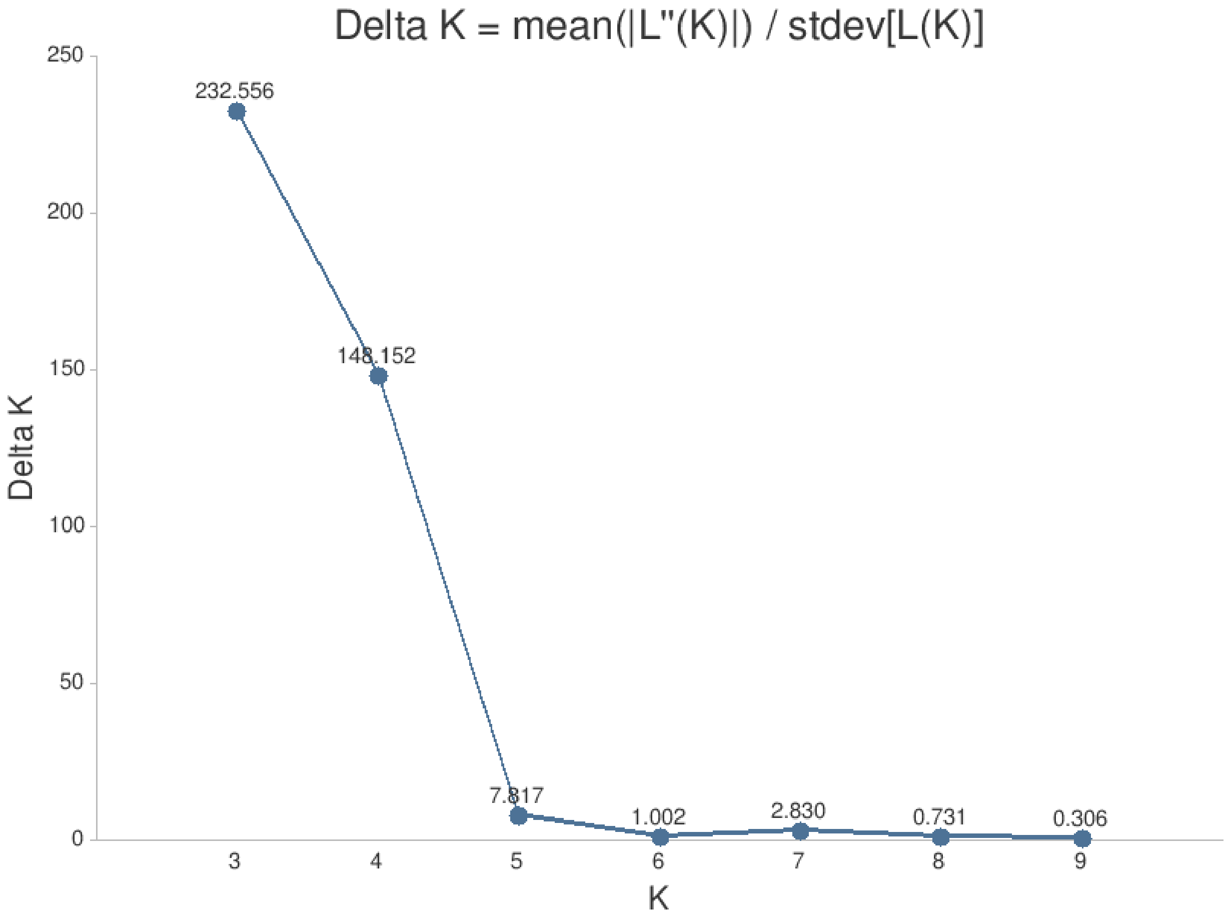

Best K By Evanno-Delta K By K graph.

The graph shows the best K equal to 3 according to delta K as proposed by Evanno.

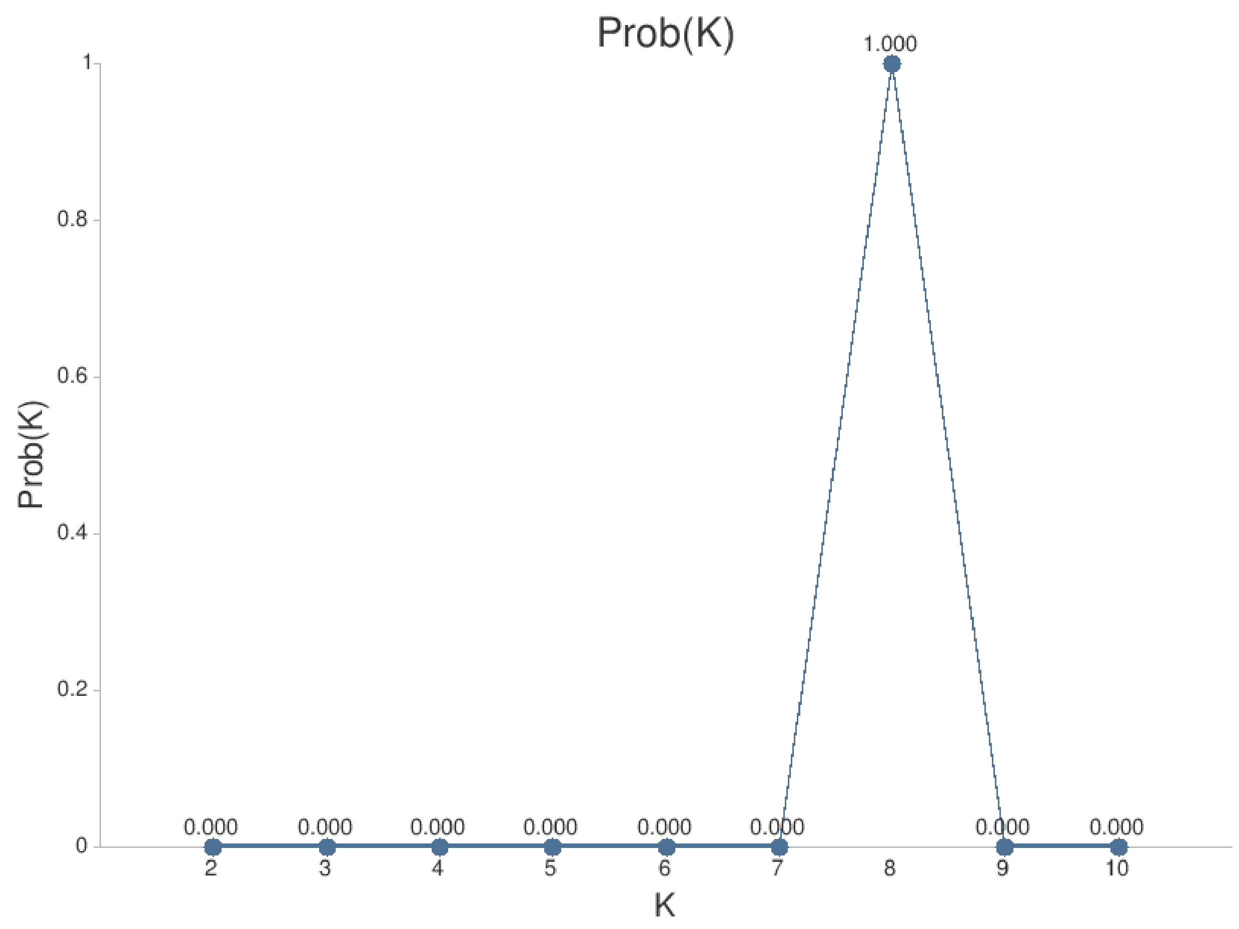

Figure S1: Highest K value by Pitchard

The graph shows the highest K value equal to 8 according to probability mesured by Pitchard method

CLUMPAK Distruct for many K's - Job 1669709181 summary

Distruct output images:

K=2 run-1-f

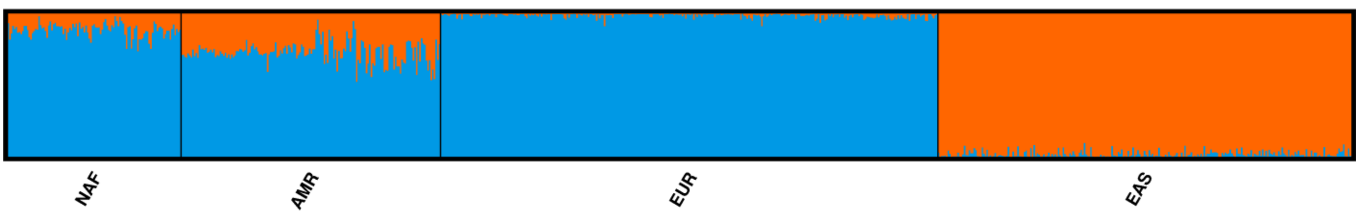

K=2 run-2-f

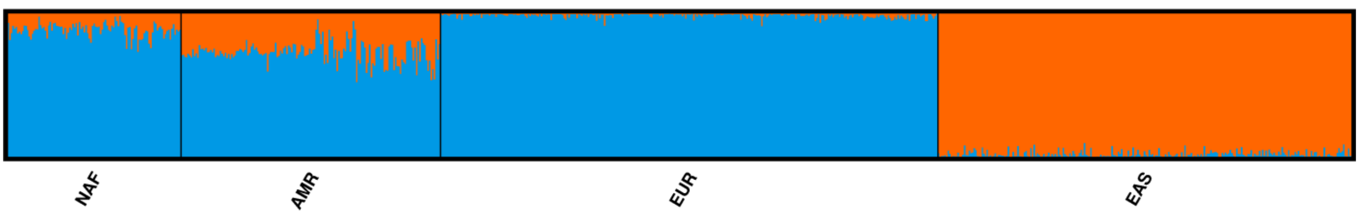

K=2 run-3-f

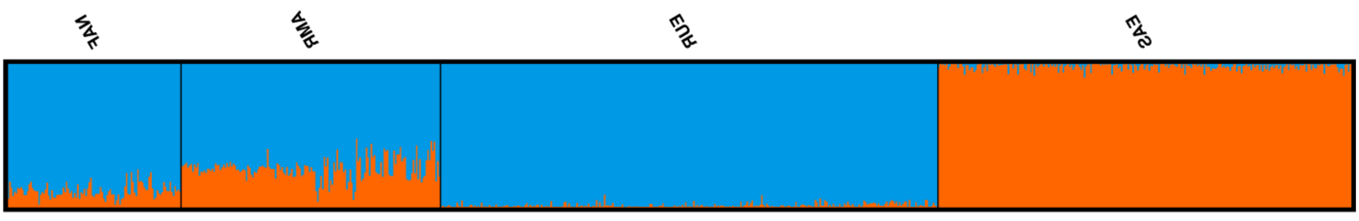

K=3 run-4-f

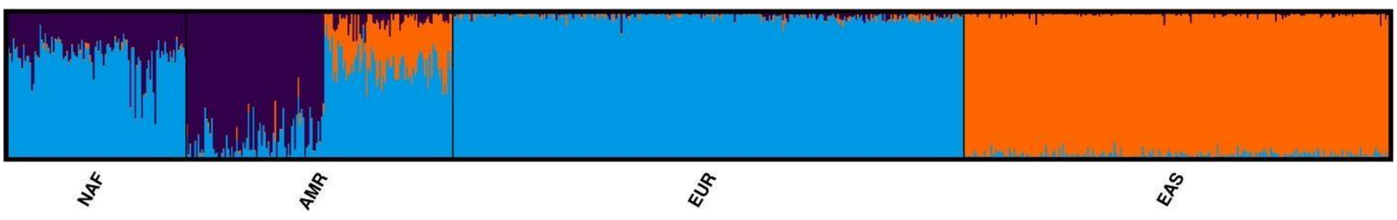

K=3 run-5-f

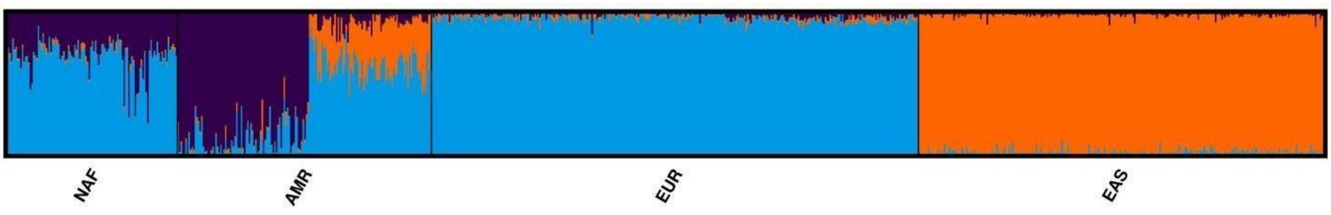

K=3 run-6-f

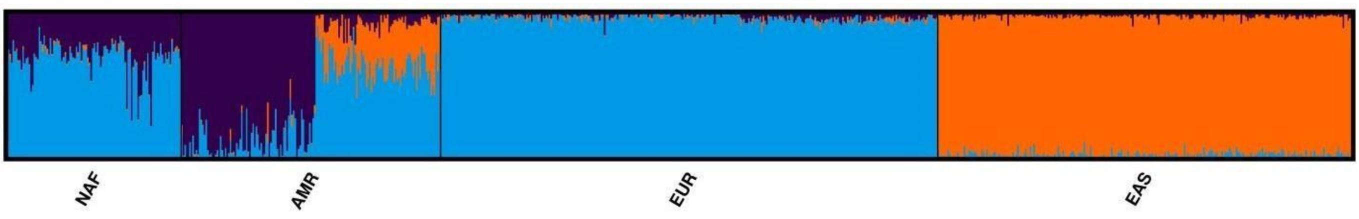

K=4 run-7-f

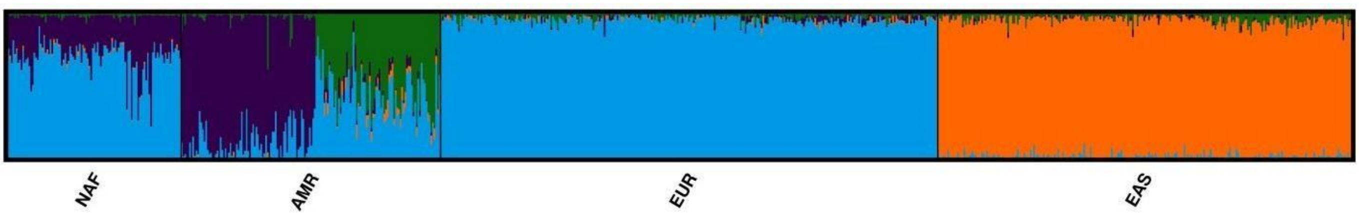

K=4 run-8-f

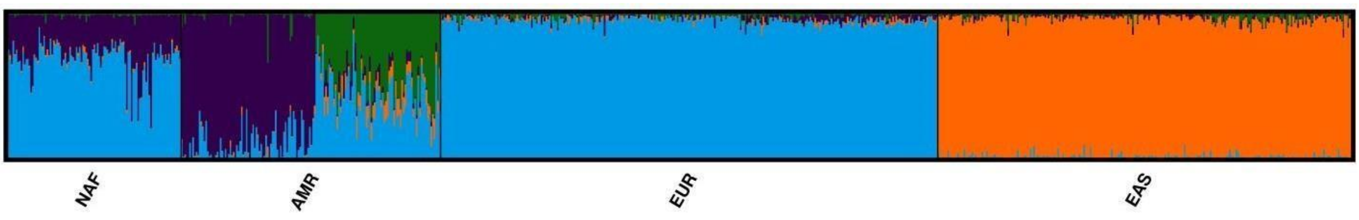

K=4 run-9-f

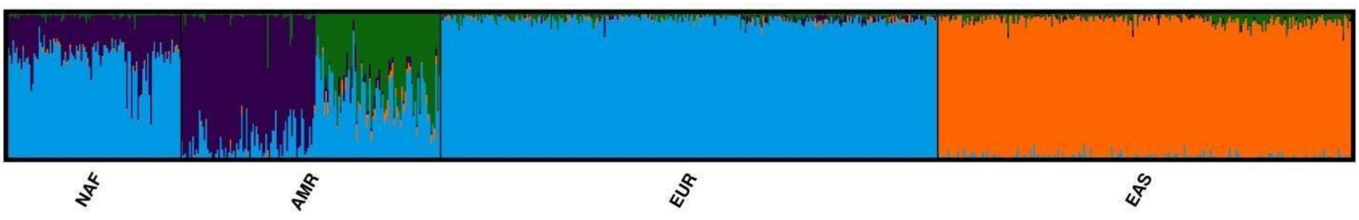

K=5 run-10-f

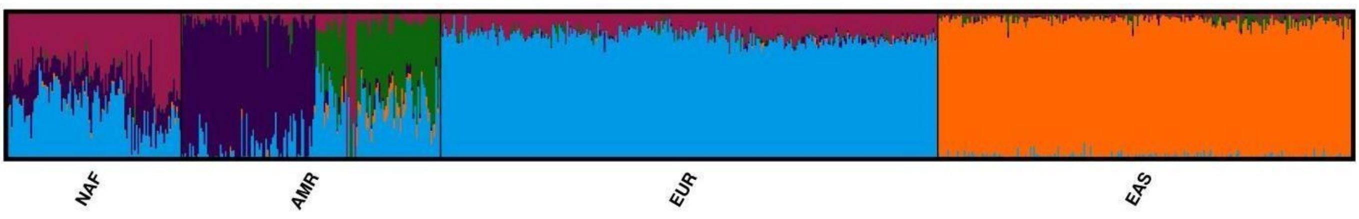

K=5 run-11-f

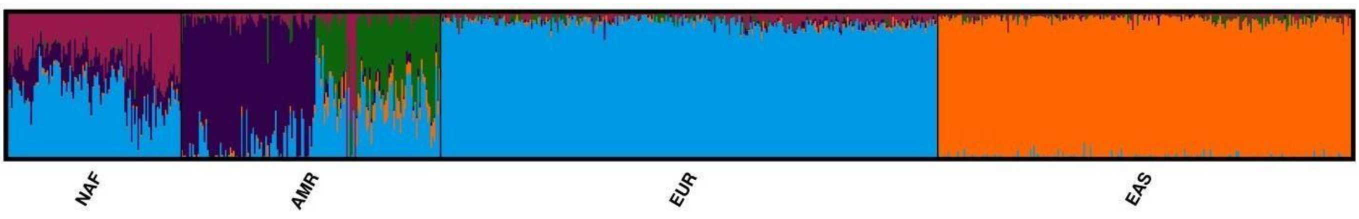

K=5 run-12-f

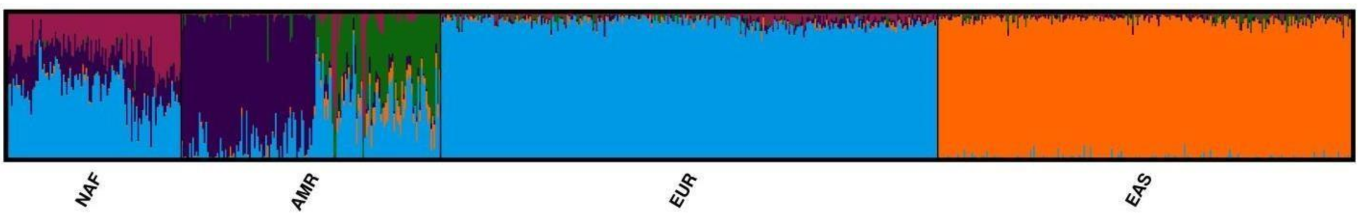

K=6 run-13-f

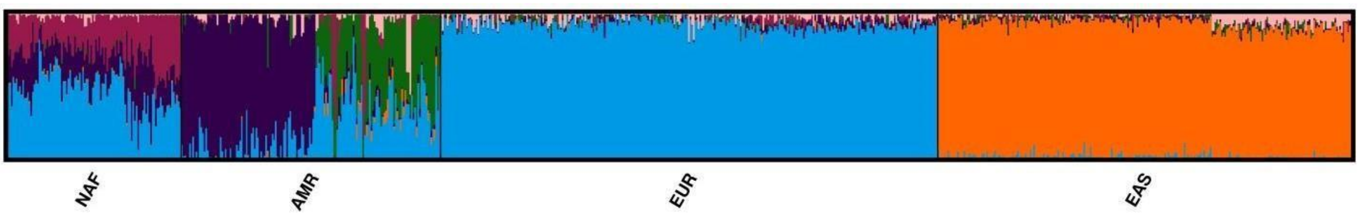

K=6 run-14-f

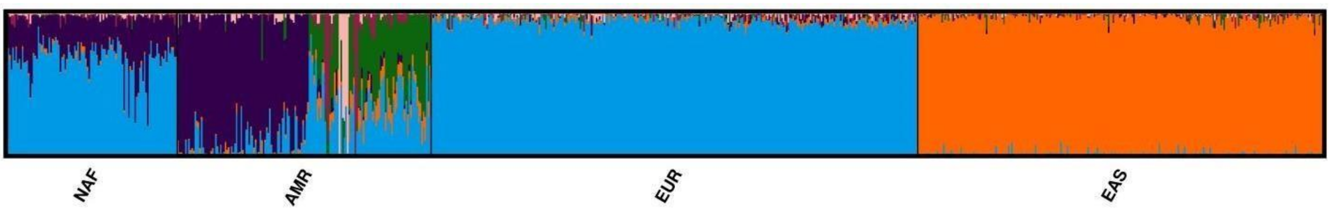

K=6 run-15-f

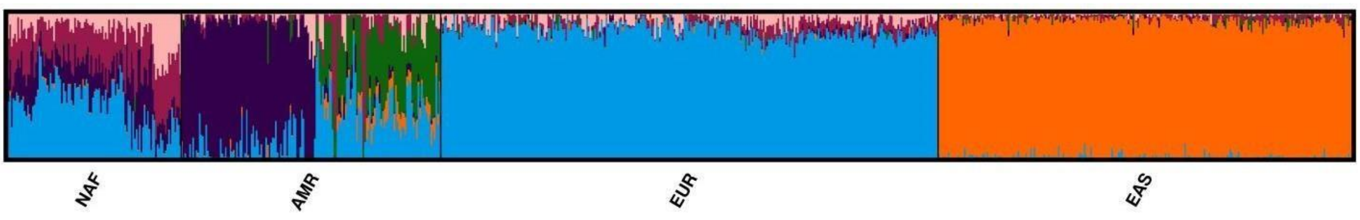

K=7 run-16-f

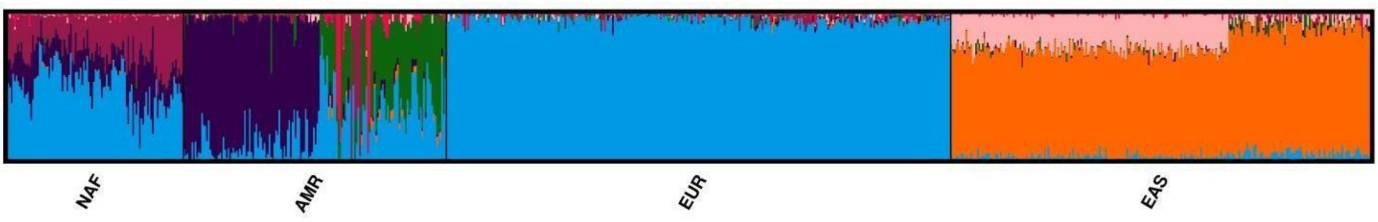

K=7 run-17-f

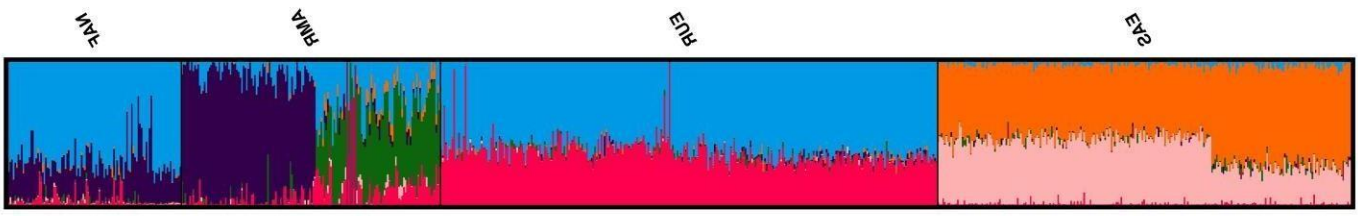

K=7 run-18-f

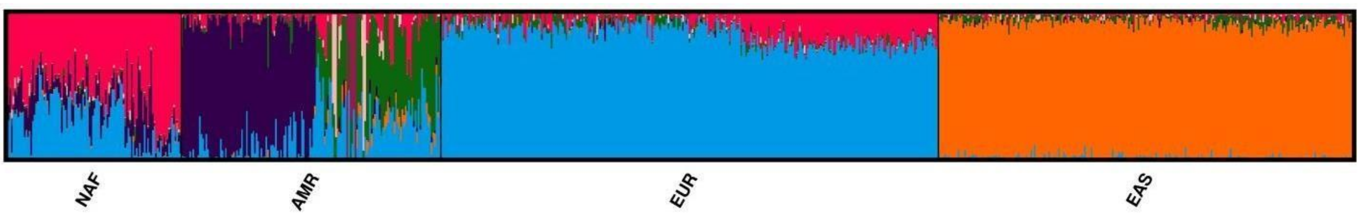

K=8 run-19-f

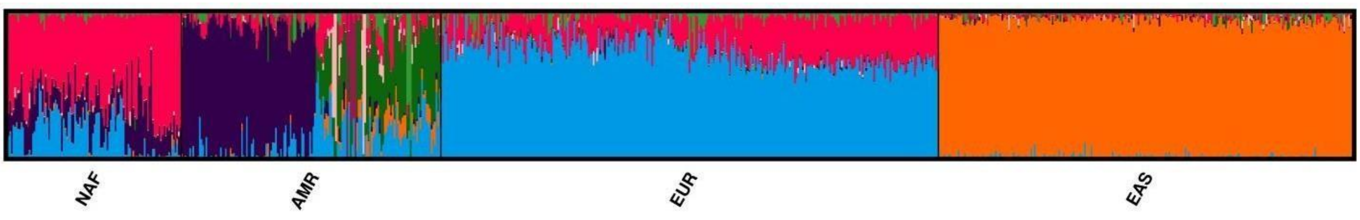

K=8 run-20-f

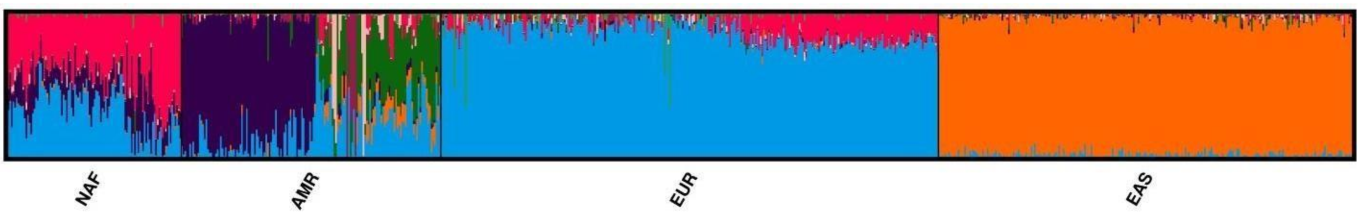

K=8 run-21-f

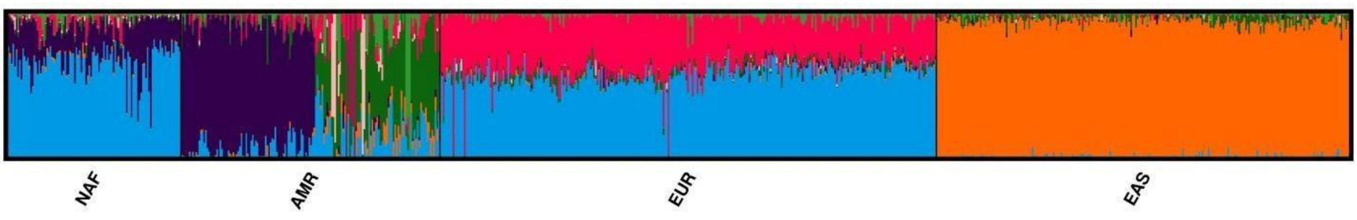

K=9 run-22-f

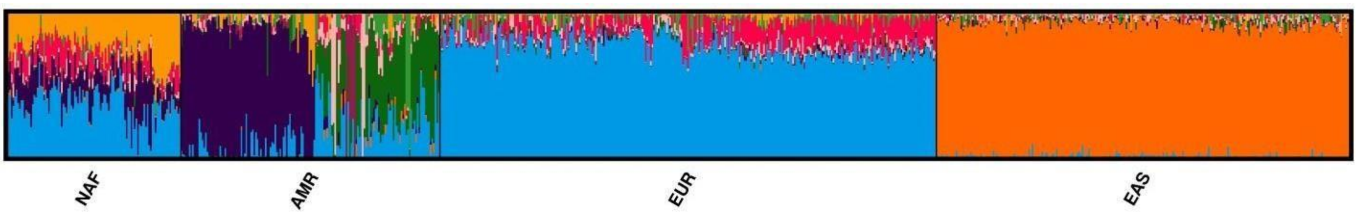

K=9 run-23-f

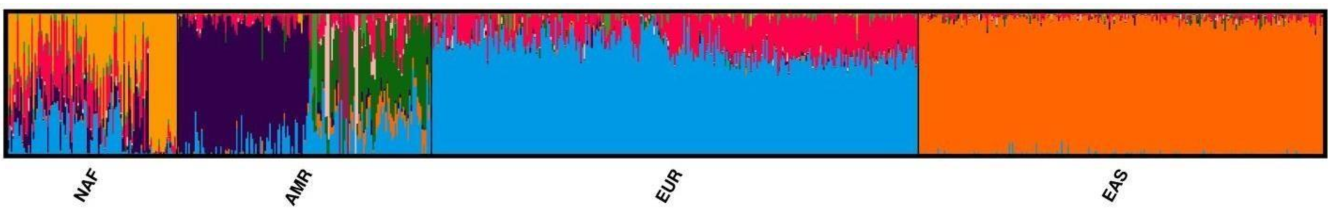

K=9

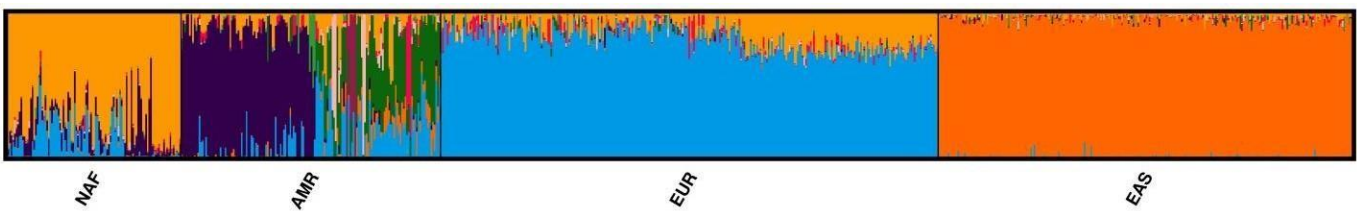

K=10 run-25-f

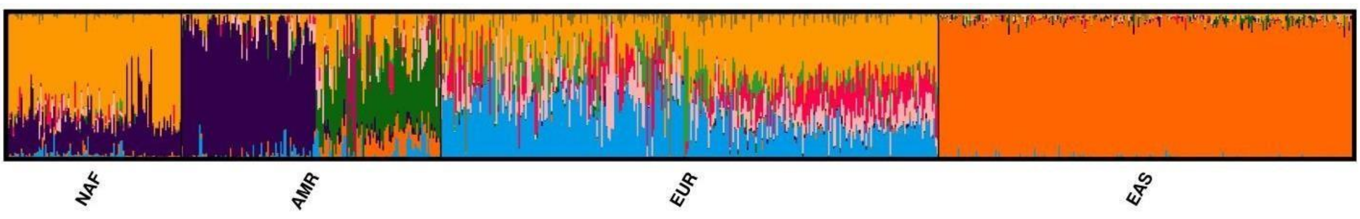

K=10 run-26-f

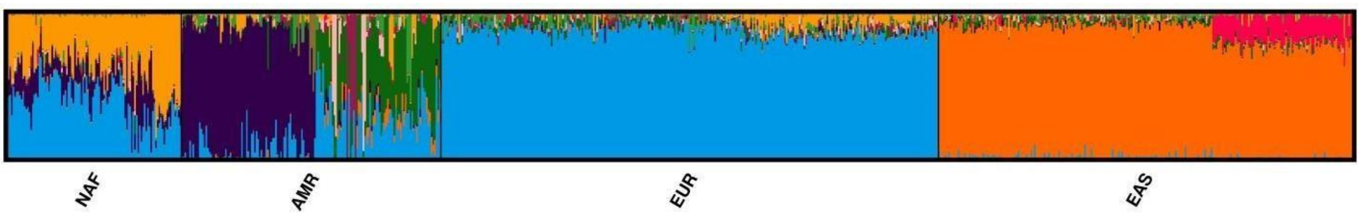

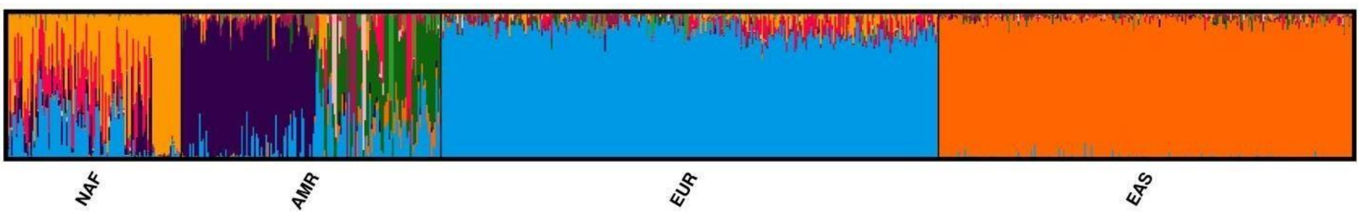

K=10 run-27-f
